# Acute renal response to changes in carbon dioxide in mechanically ventilated female pigs

**DOI:** 10.14814/phy2.70042

**Published:** 2024-09-18

**Authors:** T. Pozzi, R. V. Nicolardi, A. Fioccola, I. Fratti, F. Romitti, M. Busana, F. Collino, S. Gattarello, J. Wieditz, P. Caironi, O. Moerer, M. Quintel, K. Meissner, L. Camporota, L. Gattinoni

**Affiliations:** ^1^ Department of Anesthesiology University Medical Center Göttingen Göttingen Germany; ^2^ Department of Health Sciences University of Milan Milan Italy; ^3^ IRCCS San Raffaele Scientific Institute Milan Italy; ^4^ Department of Health Sciences, Section of Anaesthesiology, Intensive Care and Pain Medicine University of Florence Florence Italy; ^5^ Department of Surgical Sciences University of Turin Turin Italy; ^6^ Department of Medical Statistics University Medical Center Göttingen Göttingen Germany; ^7^ Department of Anesthesia and Critical Care San Luigi Gonzaga Hospital Orbassano, Turin Italy; ^8^ Department of Oncology University of Turin Turin Italy; ^9^ Department of Adult Critical Care Guy's and St Thomas' NHS Foundation Trust London UK; ^10^ Centre for Human & Applied Physiological Sciences School of Basic & Medical Biosciences, King's College London London UK

**Keywords:** acid–base, mechanical ventilation, PEEP, renal compensation, Stewart's model

## Abstract

Kidney response to acute and mechanically induced variation in ventilation associated with different levels of PEEP has not been investigated. We aimed to quantify the effect of ventilatory settings on renal acid–base compensation. Forty‐one pigs undergoing hypo‐ (<0.2 Lkg^−1^ min^−1^, PEEP 25 cmH_2_O), intermediate (0.2–0.4 Lkg^−1^ min^−1^ with either PEEP 5 or 25 cmH_2_O), or hyper‐ventilation (>0.4 Lkg^−1^ min^−1^, PEEP 5 cmH_2_O) for 48 h were retrospectively included. The decrease in pH paralleled the decrease in plasma strong ion difference (SID) in hyper‐ and intermediately ventilated groups with lower PEEP. In contrast, the plasma SID remained nearly constant in hypo‐ and intermediately ventilated groups with higher PEEP. Changes in plasma chloride concentration accounted for the changes in plasma SID (conditional *R* ^2^ = 0.86). The plasma SID changes were paralleled by mirror changes in urinary SID. Higher PEEP (25 cmH_2_O), compared to lower PEEP (5 cmH_2_O) dampened or abolished the renal compensation through its effect on hemodynamics (higher central venous and mean pulmonary pressures), irrespective of minute ventilation. During mechanical ventilation, the compensatory renal response to respiratory derangement is immediate and progressive but can be dampened by high PEEP levels.

## 1 INTRODUCTION

The renal response to ventilatory derangements has traditionally been attributed to an increase or decrease in urinary excretion of bicarbonate (HCO_3_^−^) and hydrogen ion (H^+^) (Zadek et al., 2024; Rose & Post, 2001); this interpretation refers to the conventional *bicarbonate‐centered approach* to the interpretation of acid–base disorders and their regulation. Less attention has been devoted to changes in urinary electrolytes as a renal compensation in response to alterations of blood pH (Gattinoni et al., 2006). According to Stewart's acid–base model, hydrogen ion and bicarbonate concentration are dependent variables regulated by strong ion difference (SID), partial pressure of carbon dioxide (PCO_2_), and the concentration of noncarbonic weak acids (A_TOT_) (Kellum & Elbers, 2002). Accordingly, changes in [H^+^] and [HCO_3_^−^] secondary to minute ventilation adjustment should depend on PCO_2_ level, while renal compensatory mechanisms aim to restore blood pH and should be effective through changes in plasma [SID]. However, it is unclear whether such renal responses to changes in PCO_2_ occur acutely or more slowly during respiratory alkalosis or acidosis. In addition, the potential importance of positive end‐expiratory pressure (PEEP) beyond minute ventilation on these renal compensatory mechanisms has not yet been investigated. In this study, we retrospectively investigated the kidney response to respiratory alkalosis and acidosis over a 48‐h period, aiming to (1) evaluate the timing of the compensatory renal response; (2) analyze the relationship between the urinary SID (estimated by the urinary anion gap) and plasma SID, and (3) evaluate the role of low or high PEEP on the compensatory renal response.

## 2 MATERIALS AND METHODS

This is a retrospective analysis of a previously published experimental study about the effects of mechanical power (MP)—defined as the energy delivered in 1 min to the respiratory system—on ventilator‐induced lung injury (VILI) (Vassalli et al., 2020). A more detailed description of the study protocol is reported in the Supplemental Material (10.6084/m9.figshare.24271090). Forty‐two female domestic piglets (body weight: 24.6 [22.6–25.8] kg) were maintained under total intravenous general anesthesia with propofol, sufentanyl, and midazolam and mechanically ventilated in volume‐controlled ventilation in their natural prone position for 48 h. Before the beginning of the experimental phase, animals were instrumented with esophageal balloon, central venous, pulmonary arterial, femoral arterial, and urinary catheters to monitor partitioned respiratory mechanics, gas exchange, hemodynamics and plasma and urinary acid–base variables, along with variables on renal function. One pig was excluded from the analysis due to missing plasma samples for measurements of acid–base. Therefore, 41 pigs were included. The study was approved by local authorities (18/2795, LAVES, Oldenburg, Niedersachsen, Germany).

### 2.1 Experimental protocol

After induction of general anesthesia, pigs were ventilated with 8 mL kg^−1^ of tidal volume (V_T_), a respiratory rate (RR) set to obtain an end‐tidal partial pressure of carbon dioxide (EtCO_2_) of 50 mmHg, an inspired oxygen fraction (FiO_2_) of 0.40, and a PEEP of 5 cmH_2_O. After instrumentation and a baseline recording, pigs were randomly allocated to receive low (15 J min^−1^) or high MP (30 J min^−1^) with different mixtures of V_T_, RR, and PEEP for 48 h. At the end of the experimental phase, animals were sacrificed by pentobarbital and potassium chloride injection.

### 2.2 Measurements

At baseline and after 0.5, 6, 12, 18, 24, 30, 36, 42, and 48 h from the beginning of the experimental phase (i.e., the application of the experimental ventilatory setting and the assigned MP), arterial and mixed venous blood gas analysis was performed and respiratory mechanics, gas exchange, metabolic and hemodynamic data were obtained. The complete set of measurements and calculations is listed in the Supplemental Material (10.6084/m9.figshare.24271090). Plasma and urinary electrolytes, as well as plasma creatinine and urea concentrations, were measured at baseline and after 6, 12, 24, and 48 h from the beginning of the experimental phase.

Plasma SID ([SID]) was estimated as

$$\text{Plasmatic}\;\left[\mathrm{SID}\right]=\left[Na^{+}\right]+\left[K^{+}\right]-\left[Cl^{-}\right]-\left[{\mathrm{Lac}}^{-}\right],$$

where [Na^+^], [K^+^], [Cl^−^], and [Lac] are plasma concentrations of sodium, potassium, chloride, and lactate, respectively.

Urinary SID was estimated as urinary anion gap (Gattinoni et al., 2006)

$$\text{Urinary}\;\left[\mathrm{SID}\right]=u\left[Na^{+}\right]+u\left[K^{+}\right]-u\left[Cl^{-}\right].$$

### 2.3 Study groups according to ventilation setting

To investigate the effects of a sustained PCO_2_ change on acid–base and to isolate the effect of alveolar ventilation and the application of intrathoracic‐positive pressure by PEEP, we retrospectively divided the study population according to the minute ventilation and the level of PEEP applied during the experimental phase. By doing so, four ventilation groups were obtained: a group ventilated with low ventilation (0.0–0.2 L min^−1^ kg^−1^) and high PEEP (25 cmH_2_O; L_VENT_‐H_PEEP_, 7 pigs—17%); a group receiving an intermediate level of ventilation (0.2–0.4 L min^−1^ kg^−1^) and a high PEEP (25 cmH_2_O; I_VENT_‐H_PEEP_, 7 pigs—17%); a group receiving an intermediate level of ventilation (0.2–0.4 L min^−1^ kg^−1^) and a low PEEP (5 cmH_2_O; I_VENT_‐L_PEEP_, 11 pigs—26%); and group who received high ventilation (>0.4 L min^−1^ kg^−1^) and a low PEEP (5 cmH_2_O; H_VENT_‐L_PEEP_, 17 pigs—40%).

### 2.4 Renal response to PCO_2_ variations

We expressed the relationship between acute changes in PCO_2_ and the magnitude of the compensatory renal response as *renal compensation ratio* (RCR), defined as the ratio between the difference in plasma SID (Δ[SID]) and the difference in arterial PCO_2_ (ΔPCO_2_). This ratio expresses the change in [SID] for every mmHg of PCO_2_ variation:

$${\mathrm{RCR}}_{\mathrm{PC}O_{2}}=\frac{∆\left[\mathrm{SID}\right]\left(\mathrm{mEq}\;L^{-1}\right)}{∆{\mathrm{PCO}}_{2}\left(\text{mmHg}\right)}.$$

### 2.5 Statistical analysis

Continuous variables are reported as median [interquartile range], while categorical variables are reported as percentage (number). Comparisons of continuous variables among ventilation groups at baseline and at the end of the experiment were assessed by one‐way analysis of variance (ANOVA) or Kruskal–Wallis test, as appropriately determined by Shapiro‐Wilks test and visual inspection of residual plots. Whenever possible, comparisons of continuous variables among ventilation groups within time were assessed by a two‐way ANOVA for repeated measures, with ventilation groups and time as *between* and *within* fixed effect, and animals as random effect; *post hoc* analyses were performed by adjusted pairwise comparisons after Bonferroni's correction. To model the effect of the change in electrolytes on the change in SID, a mixed linear effect model with pigs as random effect was employed; R^2^ was calculated according to Nakagawa and Schielzeth's (Nakagawa & Schielzeth, 2013). The global level of significance was set to 5%.

## 3 RESULTS

Respiratory mechanics, gas exchange, hemodynamics, and acid–base variables at baseline were similar among the four groups (see Table S1). The median [IQR] values of respiratory mechanic, gas exchange, hemodynamic, acid balance, and renal function variables after 0.5, 6, and 48 h from the beginning of the experimental phase are reported in Table 1.

**TABLE 1:** Annotations as in Figure 1. Time course of the most relevant acid–base, hemodynamic, respiratory, and gas exchange variables. Differences among groups within time were assessed by a two‐way repeated measures ANOVA. Dark gray columns refer to groups ventilated with higher PEEP (25 cmH_2_O), whereas light gray groups identify groups with lower PEEP (5 cmH_2_O).

	L_VENT_‐H_PEEP_17% (Giosa et al., 2021)	I_VENT_‐H_PEEP_ 17% (Giosa et al., 2021)	I_VENT_‐L_PEEP_ 24% (Lumb & Thomas, 1969)	H_VENT_‐L_PEEP_ 42% (Ramadoss et al., 2011)	*p*_*GROUP*_	*p*_*TIME*_	*p*_*INTER*_
Minute ventilation per kg, mL min^−1^ kg^−1^					***<0.001***	‐	‐
After 0.5 h	0.15 [0.14–0.15]	0.30 [0.28–0.31]*	0.25 [0.22–0.35]*	0.50 [0.48–0.56]*,^°^,^§^			
Arterial pH					***<0.001***	***<0.001***	***<0.001***
After 0.5 h	7.38 [7.33–7.42]	7.58 [7.54–7.62]*	7.66 [7.64–7.68]*,^°^	7.80 [7.76–7.80]*,^°^,^§^			
After 6 h	7.27 [7.25–7.31]	7.58 [7.57–7.71]*	7.67 [7.63–7.69]*	7.80 [7.78–7.80]*,^°^,^§^			
After 48 h	7.22 [7.22–7.26]	7.47 [7.43–7.54]*	7.53 [7.51–7.55]*	7.60 [7.56–7.63]*,^°^,^§^			
PaCO_2_, mmHg					***<0.001***	*0.615*	***<0.001***
After 0.5 h	51 [50–56]	31 [30–35]*	29 [27–30]*	15 [15–20]*,^°^,^§^			
After 6 h	60 [55–65]	30 [28–33]*	26 [24–30]*	14 [11–16]*,^°^,^§^			
After 48 h	60 [55–69]	35 [33–36]*	24 [21–28]*	13 [12–17]*,^°^,^§^			
Plasma [Na^+^], mEq L^−1^					*0.191*	***<0.001***	*0.424*
Baseline	144 [142–145]	145 [144–145]	143 [141–145]	145 [144–147]			
After 6 h	147 [147–148]	147 [146–147]	146 [142–148]	146 [143–149]			
After 48 h	148 [146–148]	148 [146–149]	146 [144–147]	148 [146–150]			
Plasma [K^+^], mEq L^−1^					***<0.001***	***<0.001***	***<0.001***
Baseline	4.0 [3.9–4.2]	4.0 [3.9–4.4]	3.9 [3.8–4.3]	4.0 [3.9–4.5]			
After 6 h	5.1 [5.0–5.2]	4.6 [4.3–5.0]	4.6 [4.4–5.2]	4.1 [4.0–4.5]*			
After 48 h	7.1 [5.9–7.1]	5.5 [4.6–5.7]	3.9 [3.7–4.0]*, ^°^	3.8 [3.5–4.0]*, ^°^			
Plasma [Cl^−^], mEq L^−1^					***<0.001***	***<0.001***	***<0.001***
Baseline	105 [104–106]	107 [104–107]	104 [101–104]	104 [103–107]			
After 6 h	110 [108–112]	110 [109–113]	110 [106–111]	112 [111–115]			
After 48 h	111 [108–112]	113 [113–116]	119 [117–120]*,^°^	124 [123–126]*,^°^,^§^			
Plasma [Lac], mEq L^−1^					***0.007***	***<0.001***	*0.067*
Baseline	0.5 [0.4–0.6]	0.7 [0.3–1.4]	0.5 [0.5–0.6]	0.5 [0.4–0.7]			
After 6 h	2.1 [0.7–2.6]	1.4 [1.1–1.8]	0.9 [0.7–0.9]	1.6 [1.2–2.0]			
After 48 h	1.0 [0.6–1.0]	1.0 [1.0–1.1]	0.5 [0.4–0.7]	1.0 [0.9–1.3]			
Plasma [SID], mEq L^−1^					***<0.001***	***<0.001***	***<0.001***
Baseline	43.6 [41.7–43.9]	42.7 [41.4–43.9]	43.2 [41.8–44.7]	46.1 [44.4–46.5]			
After 6 h	42.5 [40.3–43.1]	40.5 [39.4–42.7]	40.8 [39.8–41.6]	36.6 [35.7–38.2]*,^°^,^§^			
After 48 h	43.7 [42.0–45.4]	39.2 [38.8–40.6]	31.8 [30.1–32.2]*,^°^	27.4 [25.2–29.6]*,^°^,^§^			
Urinary [SID], mEq L^−1^					*0.162*	*0.082*	***0.005***
Baseline	42 [37–96]	76 [71–120]	62 [38–95]	44 [27–69]			
After 6 h	53 [37–74]	77 [64–129]	54 [42–97]	114 [64–145]			
After 48 h	49 [40–59]	125 [111–132]	69 [46–132]	113 [61–146]*			
Cumulative fluid intake, L					***<0.001***	***<0.001***	*0.096*
After 0.5 h	1.8 [1.6–1.9]	1.8 [1.4–2.0]	1.2 [0.9–1.4]	1.6 [1.3–2.2]			
After 6 h	3.8 [3.6–4.2]	3.8 [2.8–4.0]	2.0 [1.6–2.0]	2.9 [2.4–3.3]			
After 48 h	10.5 [9.2–12.4]	10.8 [9.3–11.1]	8.6 [7.9–9.0]	9.0 [8.4–10.6]			
Cumulative urine output, L					***<0.001***	***<0.001***	***<0.001***
After 0.5 h	0.1 [0.1–0.3]	0.1 [0.1–0.2]	0.2 [0.1–0.3]	0.2 [0.1–0.5]			
After 6 h	0.3 [0.2–0.6]	0.4 [0.3–0.6]	0.8 [0.7–1.0]	0.7 [0.5–1.3]			
After 48 h	0.7 [0.4–1.3]	1.1 [0.9–1.8]	2.8 [2.0–3.5]*,^°^	3.0 [2.2–3.7]*, ^°^			
Fluid balance, L					***<0.001***	***<0.001***	***<0.001***
After 0.5 h	1.6 [1.2–1.7]	1.5 [1.3–1.8]	0.9 [0.8–1.1]	1.4 [1.0–1.9]			
After 6 h	3.3 [3.2–3.5]	3.3 [2.2–3.6]	1.1 [0.9–1.4]	1.9 [1.7–2.4]			
After 48 h	9.2 [8.2–11.5]	9.7 [7.6–10.0]	5.7 [5.0–6.6]*,^°^	6.0 [5.8–7.5]*,^°^			
Mean airway pressure, cmH_2_O					***<0.001***	*0.070*	***<0.001***
After 0.5 h	30 [29–31]	36 [36–39]*	11 [10–11]*,^°^	15 [13–16]*,^°^,^§^			
After 6 h	29 [28–31]	33 [29–36]*	11 [9–12]*,^°^	15 [13–16]*,^°^,^§^			
After 48 h	28 [28–29]	33 [32–35]*	11 [10–12]*,^°^	17 [16–20]*,^°^,^§^			
Respiratory system elastance, cmH_2_O L^−1^					***<0.001***	*0.093*	***<0.001***
After 0.5 h	74.4 [72.9–79.4]	88.1 [77.0–105.5]	34.9 [32.5–36.2]	33.9 [29.5–36.7]			
After 6 h	58.4 [53.2–61.3]	75.3 [66.1–80.9]	35.1 [33.1–39.9]	35.7 [33.9–40.2]			
After 48 h	54.9 [49.6–58.6]	74.0 [69.3–74.6]	36.7 [32.4–43.2]	47.1 [41.5–64.5]			
PaO_2_/FiO_2_, mmHg					***<0.001***	***<0.001***	***0.002***
After 0.5 h	555 [508–574]	618 [614–623]*	605 [579–622]*	625 [600–639]*			
After 6 h	460 [419–472]	573 [565–605]*	535 [508–559]*	595 [575–605]*,^§^			
After 48 h	445 [410–483]	562 [539–570]*	540 [527–571]*	588 [569–621]*,^§^			

*Note*: Bold: *p* < 0.050.

Abbreviations: [Cl^−^], chloride concentration; [K^+^], potassium concentration; [Lac^−^], lactate concentration; [Na^+^], sodium concentration; FiO_2_, inspired oxygen fraction; PaCO_2_, arterial carbon dioxide partial pressure; PaO_2_, arterial oxygen partial pressure; SID, strong ion difference.

* *p*<0.05 vs L_VENT_‐H_PEEP_;

^°^ *p*<0.05 vs I_VENT_‐H_PEEP_;

^§^ *p*<0.05 vs I_VENT_‐L_PEEP_.

### 3.1 Time course of minute ventilation, PaCO_2_, and arterial pH

**FIGURE 1:**
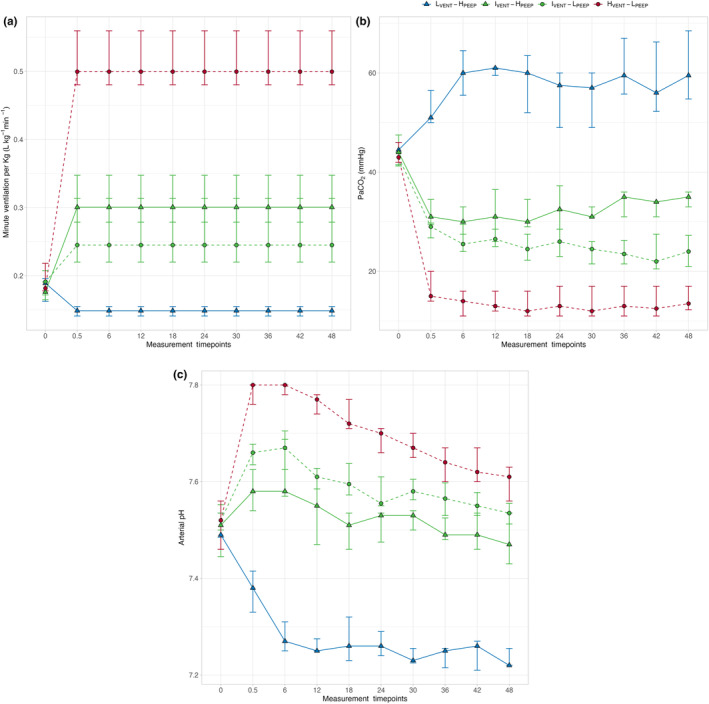
Symbols and annotations: L_VENT_‐H_PEEP_, low‐minute ventilation high PEEP (blue triangles); I_VENT_‐H_PEEP_, intermediate‐minute ventilation high PEEP (green triangles); I_VENT_‐L_PEEP_, intermediate‐minute ventilation low PEEP (green circles); H_VENT_‐L_PEEP_, high‐minute ventilation low PEEP (red circles). The dashed and the solid lines connects groups treated with 5 or 25 cmH_2_O of PEEP, respectively. Note that, for the sake of clarity, the intervals between measurements at baseline and 0.5 h has been arbitrarily equated to an interval of 6 h. Differences among groups within time were assessed by a two‐ways repeated measures ANOVA. (a) Time course of minute ventilation in the four experimental groups. Minute ventilation was significantly different among low, intermediate, and high ventilation groups (*p*_*GROUP*_ <0.001) during the experimental phase (from 0.5 to 48 h) due to the experimental protocol. No significant differences were found between intermediate groups ventilated with higher and lower PEEP (*p* = 0.321). (b) Time course of arterial carbon dioxide partial pressure (PaCO_2_) in the four experimental groups. Arterial PaCO_2_ behavior was significantly different among low, intermediate, and high ventilation groups; of note, arterial PCO_2_ began to be significantly different also between intermediately ventilated groups treated with higher or lower PEEP after 24 h (*p* < 0.050). *p*_*GROUP*_ <0.001, *p*_*TIME*_ = 0.615, *p*_*INTER*_ <0.001. (c) Time course of arterial pH in the four experimental groups. Arterial pH behavior was significantly different among low, intermediate, and high ventilation groups within time (*p*_*INTER*_ <0.001). After reaching the near‐PCO_2_ steady state (0.5–6 h), arterial pH significantly decreased with time in high and intermediate ventilation groups, while it remained unchanged in the low ventilation group. *p*_*GROUP*_ <0.001, *p*_*TIME*_ <0.001, *p*_*INTER*_ <0.001.

In Figure 1, Panel A, we report the minute ventilation applied to the different groups. As shown, minute ventilation was kept constant in each group throughout the experiment. The arterial PCO_2_ (PaCO_2_) time course is presented in Panel B; in the hyper‐ and intermediately ventilated groups (i.e., H_VENT_‐L_PEEP_, I_VENT_‐H_PEEP_, and I_VENT_‐L_PEEP_), PaCO_2_ sharply decreased within about 30 min, reaching a near‐steady state. In contrast, in the hypo‐ventilated group (L_VENT_‐H_PEEP_), the near‐steady state was reached after 6 h. Of note, in the groups with intermediate ventilation, PaCO_2_ was not significantly different in the first 24 h; in the remaining experimental time, PaCO_2_ became significantly higher in the group with higher PEEP, while it tended to decrease in the group with lower PEEP. Panel C shows the pH time course in the four groups. As shown, although the pH initially changed in accordance with the change in PaCO_2_, it then tended toward baseline values in the highly and intermediately ventilated group. In contrast, the pH remained unchanged in the low ventilation group with higher PEEP.

### 3.2 Renal compensation

**FIGURE 2:**
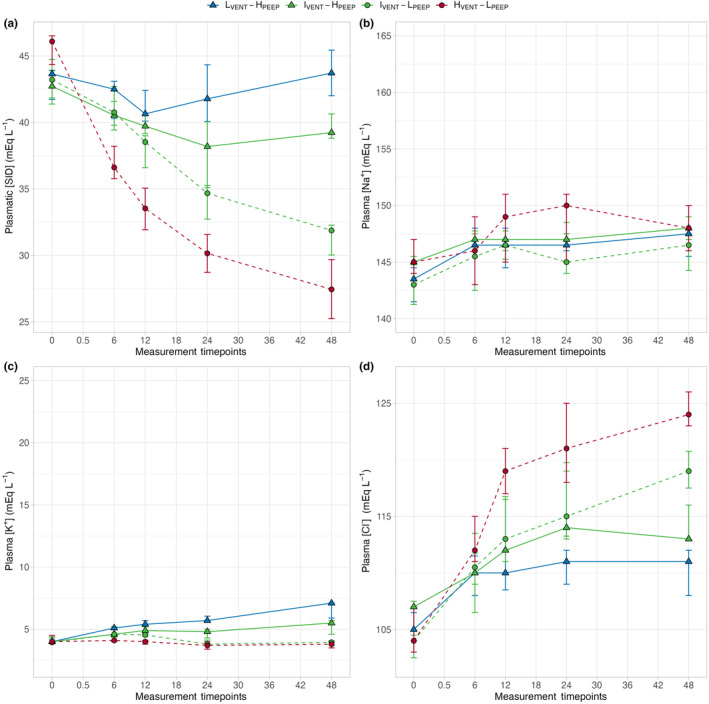
Symbols and annotations as in Figure 1. Differences among groups within time were assessed by a two‐ways repeated measures ANOVA. (a) Time course of plasma strong ion difference (plasma [SID]) in the four experimental groups. Plasma [SID] showed a significant interaction among groups within time, resulting in statistically different plasma SID among all the four groups at 48 h. *p*_*GROUP*_ <0.001, *p*_*TIME*_ <0.001, *p*_*INTER*_ <0.001. (b) Time course of plasma sodium concentration (plasma [Na^+^]) in the four experimental groups. All four experimental groups showed a similar behavior within time. *p*_*GROUP*_ = 0.191, *p*_*TIME*_ <0.001, *p*_*INTER*_ = 0.424. (c) Time course of plasma potassium concentration (plasma [K^+^]) in the four experimental groups. The lower (dashed lines) and higher (solid lines) PEEP groups showed a significantly different behavior within time. *p*_*GROUP*_ <0.001, *p*_*TIME*_ <0.001, *p*_*INTER*_ <0.001. (d) Time course of plasma chloride concentration (plasma [Cl^−^]) in the four experimental groups. The lower (dashed lines) and higher (solid lines) PEEP groups showed a significantly different behavior within time. *p*_*GROUP*_ <0.001, *p*_*TIME*_ <0.001, *p*_*INTER*_ <0.001.

**FIGURE 3:**
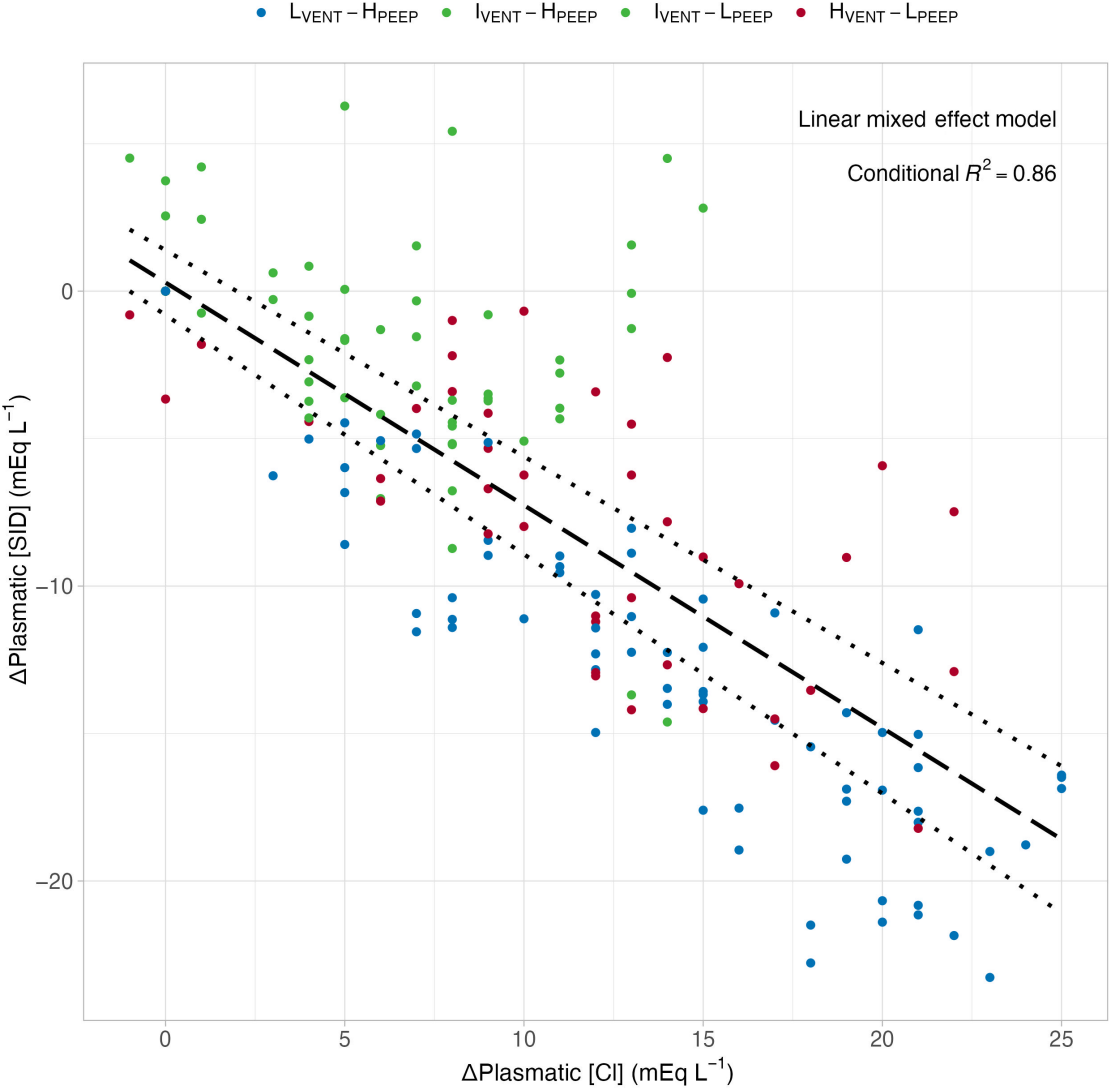
Relationship between changes in plasma SID (ΔPlasma [SID]) and changes in plasma chloride concentration (ΔPlasma [Cl^−^]). A linear mixed effect model was used to model the relationship: $\Delta \text{Plasmatic}\;\left[\mathrm{SID}\right]=0.29-0.76\times \Delta \text{Plasmatic}\;\left[Cl^{-}\right]$, *p* < 0.001, Conditional *R* ^2^ = 0.86. Dotted lines represent 0.95% confidence intervals of the model. L_VENT_‐H_PEEP_, low‐minute ventilation high PEEP (blue dots); I_VENT_‐H_PEEP_, intermediate‐minute ventilation high PEEP (green dots); I_VENT_‐L_PEEP_, intermediate‐minute ventilation low PEEP (green dots); H_VENT_‐L_PEEP_, high‐minute ventilation low PEEP (red dots).

*Strong ion difference in plasma*. As shown in Figure 2 Panel A, plasma [SID] significantly decreased in two groups treated with lower PEEP, while it did not change in both the groups with higher PEEP, irrespective of minute ventilation. The time course of its main determinants (i.e., sodium, potassium, and chloride) is presented in Panels B–D. As shown, sodium behaved similarly in the different groups, whereas the time course of potassium and chloride concentrations were remarkably different. Indeed, potassium significantly increased in the two groups with higher PEEP. The opposite behavior was observed for chloride: it increased significantly over time in the lower PEEP groups, while remaining essentially stable in the two higher PEEP groups. Of note, variations in plasma chloride concentration almost completely accounted for the changes in plasma [SID]. Indeed, the increases in plasma chloride concentration were associated with almost equivalent decreases in plasma [SID] (see Figure 3). Interestingly, despite receiving a slightly lower amount of fluid and, thus, chloride, the I_VENT_‐L_PEEP_ group more effectively managed to increase plasma chloride concentration with respect to I_VENT_‐H_PEEP_ (see Figure 2 and S1).

#### 3.2.1 Strong ion difference in urine

The changes in urinary SID inversely paralleled the changes in plasma [SID] (see Figure S2). Indeed, when urinary [SID] was greater than plasma [SID], the latter progressively decreased, likely due to chloride reabsorption, while when urinary [SID] did not change, as in the hypo‐ventilated group, plasma [SID] remained unchanged (≈40 mEq L^−1^).

#### 3.2.2 Quantification of renal response to acid–base variations

**FIGURE 4:**
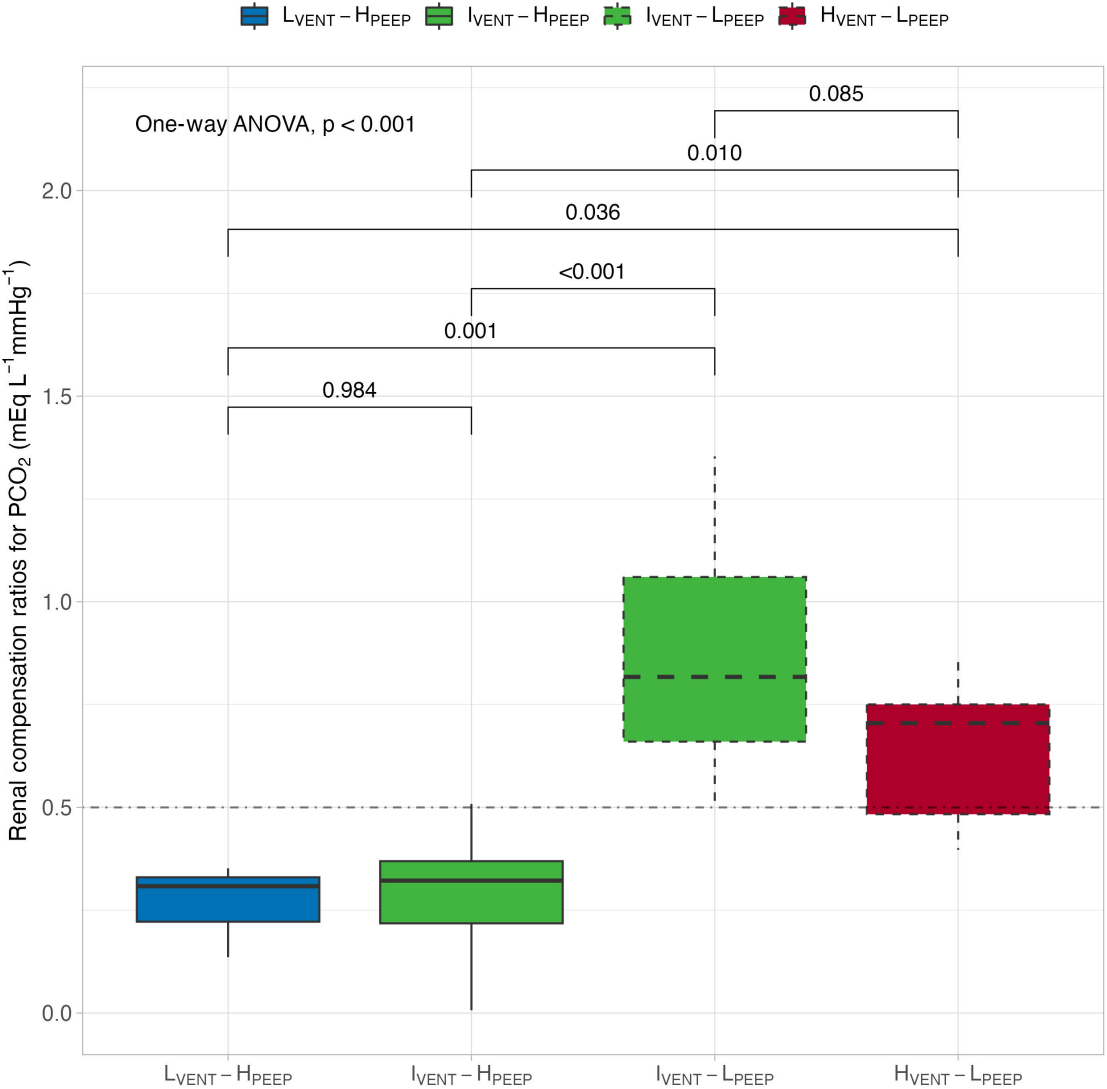
Renal compensation ratios among experimental groups after 48 h of mechanical ventilation. L_VENT_‐H_PEEP_, low‐minute ventilation high PEEP (blue, solid line); I_VENT_‐H_PEEP_, intermediate‐minute ventilation high PEEP (green, solid line); I_VENT_‐L_PEEP_, intermediate‐minute ventilation low PEEP (green, dashed line); H_VENT_‐L_PEEP_, high‐minute ventilation low PEEP (red, dashed line). The horizontal line represents the expected compensation for chronic respiratory alkalosis (Rose & Post, 2001). Differences among groups were assessed by a one‐way ANOVA, while *post hoc* pairwise comparisons were performed.

Renal compensation ratios for PaCO_2_ over the whole experimental period are presented in Figure 4. As shown, the greatest renal compensation ratios for PaCO_2_ were observed in groups with lower PEEP; indeed, every mmHg of PaCO_2_ change resulted in a change in plasma [SID] of 0.71 [0.48–0.75] mEq L^−1^ mmHg^−1^ in H_VENT_‐L_PEEP_ group and of 0.82 [0.66–1.06] mEq L^−1^ mmHg^−1^ in I_VENT_‐L_PEEP_ group. In contrast, pigs ventilated with higher PEEP showed significantly lower renal compensation ratios (−0.31[−0.33 – −0.09] mEq L^−1^ mmHg^−1^ for I_VENT_‐H_PEEP_ group and 0.322[0.218–0.369] mEq L^−1^ mmHg^−1^ for L_VENT_‐H_PEEP_ group).

### 3.3 Ventilatory setting, hemodynamics, and renal function

**FIGURE 5:**
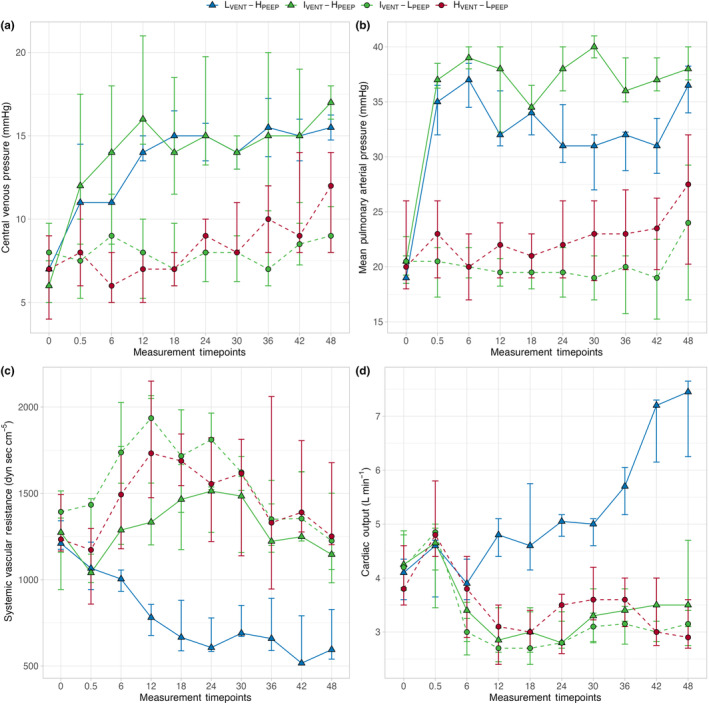
Symbols and annotations as in Figure 1. Differences among groups within time were assessed by a two‐way repeated measures ANOVA. (a) Time course of central venous pressure in the four experimental groups. Central venous pressure was significantly different among groups, specially between groups ventilated with higher and lower PEEP. *p*_*GROUP*_ <0.001, *p*_*TIME*_ <0.001, *p*_*INTER*_ = 0.401. (b) Time course of mean pulmonary arterial pressure in the four experimental groups. Mean pulmonary arterial pressure was significantly different among groups, specially between groups ventilated with higher and lower PEEP. *p*_*GROUP*_ <0.001, *p*_*TIME*_ = 0.077, *p*_*INTER*_ = 0.131. (c) Time course of systemic vascular resistance in the four experimental groups. The hypo‐ventilated group with higher PEEP showed a significantly different behavior within time compared to other groups. *p*_*GROUP*_ = 0.123, *p*_*TIME*_ = 0.007, *p*_*INTER*_ = 0.026. (d) Time course of cardiac output in the four experimental groups. The hypo‐ventilated group with higher PEEP showed a significantly different behavior within time compared to other groups. *p*_*GROUP*_ = 0.004, *p*_*TIME*_ = 0.001, *p*_*INTER*_ <0.001.

**FIGURE 6:**
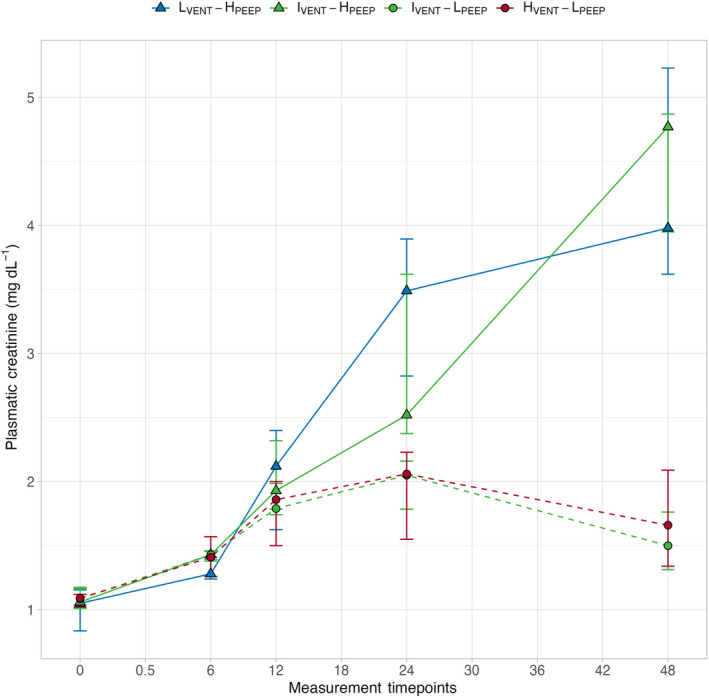
Symbols and annotations as in Figure 1. Differences among groups within time were assessed by a two‐way repeated measures ANOVA. Time course of plasma creatinine in the four experimental groups. The groups ventilated with higher PEEP showed a significantly different behavior over time compared to groups with lower PEEP. *p*_*GROUP*_ <0.001, *p*_*TIME*_ <0.001, *p*_*INTER*_ <0.001.

The time course of central venous pressure, mean pulmonary artery pressure, systemic vascular resistance and cardiac output are presented in Figure 5. As shown, central venous and mean pulmonary artery pressure were significantly higher in groups treated with higher PEEP, regardless of minute ventilation. Systemic vascular resistance was significantly lower and cardiac output was significantly higher in the low ventilation and high PEEP group compared with the other groups. The time course of plasma creatinine and blood urea nitrogen (BUN) concentrations are presented in Figure 6, Figure S3. As shown both creatinine and BUN increased significantly over time in the higher PEEP groups compared with lower PEEP groups (*p*_*INTER*_ <0.001 and *p*_*INTER*_ <0.001, respectively). (see Table 1, Table S2).

## 4 DISCUSSION

The main findings of this study are that (1) kidneys provide an immediately progressive electrolyte compensation to variation of minute ventilation; (2) the experimental observations could be explained by the physicochemical approach, which focuses on the relationship between plasma and urinary SID; (3) higher PEEP impairs renal function and the renal ability to compensate for changes in PCO_2_.

### 4.1 Study population

Our study population consisted of 41 pigs treated with different combinations of various degrees of minute ventilation and PEEP (5 vs. 25 cmH_2_O) (Vassalli et al., 2020). We arbitrarily grouped pigs according to two criteria: minute ventilation (hypo‐ventilation: <0.2 L kg^−1^ min^−1^, intermediate ventilation: 0.2–0.4 L kg^−1^ min^−1^, or hyper‐ventilation: >0.4 L kg^−1^ min^−1^) and the level of PEEP (lower: 5 vs. higher: 25 cmH_2_O). Such thresholds of minute ventilation were chosen in order to clearly identify hypercapnia, mild hypocapnia, and severe hypocapnia. These three groups were successfully defined physiologically, as shown in Figure 1a Figure S4.

### 4.2 Response to the applied minute ventilation

As shown in Figure 1b, the PaCO_2_ response to hyper‐ and intermediate ventilation (i.e., I_VENT_‐H_PEEP_, I_VENT_‐L_PEEP_, and H_VENT_‐L_PEEP_ groups) was rapid: after 30 min, PaCO_2_ was at a near‐steady state. In contrast, in the hypo‐ventilated group, the near‐equilibrium was reached after 6 h. The different rates of PaCO_2_ raise and descent to reach the near‐steady state have been already reported (Arbus et al., 1969; Giosa et al., 2021; Ivanov & Nunn, 1968; Lumb & Thomas, 1969), although the definition of steady state is somehow variable; variations of PCO_2_ limited to 1–3 mmHg—due to variation in CO_2_ stores—are usually accepted to define a steady state (Arbus et al., 1969). In our experiment, a near PCO_2_ steady state was obtained in 0.5–6 h, during which minute ventilation (see Figure 1a) and VCO_2_ (see Figure S5) did not change significantly. This near‐steady‐state condition was roughly maintained throughout the experiment in hypo‐ and hyper‐ventilated groups (L_VENT_‐H_PEEP_ and H_VENT_‐L_PEEP_ groups). In contrast, in the two groups with intermediate ventilation, the PCO_2_ increased in the higher PEEP (I_VENT_‐H_PEEP_) group and decreased in lower (I_VENT_‐L_PEEP_) PEEP group after 24 h, despite similar VCO_2_ and minute ventilation.

While PCO_2_ reached a near‐steady state in all groups, after 6 h, the pH progressively decreased toward a normal value in hyper‐ and intermediately ventilated groups (I_VENT_‐H_PEEP_, I_VENT_‐L_PEEP_, and H_VENT_‐L_PEEP_ groups), while it remained constant after reaching the near‐steady state in the hypo‐ventilated group (L_VENT_‐H_PEEP_ group; see Figure 1c). This suggests the progression of a compensatory mechanism related to the kidney, which might be explained by the relationship between plasma and urinary [SID] behavior (Gattinoni et al., 2006). Indeed, as shown in Figure 2a, plasma [SID] decreased in the hyper‐ and intermediately ventilated groups, whereas it remained essentially unchanged during hypoventilation, when it should have increased.

The reverse trend was observed In urinary [SID] (see Figure S2). Notably, in the two groups with similar ventilation (I_VENT_‐H_PEEP_, I_VENT_‐L_PEEP_ groups, intermediately ventilated), the SID reduction—that is, the renal compensation—was significantly lower in the higher PEEP group. Actually, in plasma and in urine, we only measured sodium, potassium, and chloride, whereas the other components of SID were not measured; however, the plasma and urinary anion gaps constitute the main determinants of SID and we used them as proof of concept (Kellum & Elbers, 2002).

Urinary [SID] adjustments (Figure S2) should account for the variations in plasma [SID]; however, the mechanisms through which the PCO_2_ changes induce the renal compensation are not well elucidated. The pH variations are likely the major determinant for these effects (Brown & Wagner, 2012); this is in apparent contrast to Stewart's approach: indeed, in this case, pH likely determined an SID variation and not vice versa. However, as already recognized by Stewart, the independency of SID, PCO_2_, and A_TOT_ is only valid in plasma without any organ interaction; when this is present, also SID and PCO_2_ can become interdependent through pH variations (Kellum & Elbers, 2002; Langer et al., 2015).

When we consider the behavior of SID and its components, two considerations appear evident. First, the main determinant of changes in plasma SID is the variation of chloride concentration (see Figure 2), confirming that changes in chloride concentration over a wide range are key in maintaining the acid–base homeostasis, and are otherwise harmless (Berend et al., 2012; Koch & Taylor, 1992; Ramadoss et al., 2011). In contrast, sodium concentration must be maintained within narrow ranges, as sodium is the main determinant of osmolarity and volume distribution, and so must potassium, as a key determinant in transmembrane potential regulation (Rose & Post, 2001). Second, regardless of the minute ventilation, the groups with higher PEEP (L_VENT_‐H_PEEP_ and I_VENT_‐H_PEEP_ groups) showed significantly higher SID, higher potassium, and lower chloride concentrations, particularly in the last 24 h of the experiment, as compared to lower PEEP groups (I_VENT_‐L_PEEP_ and H_VENT_‐L_PEEP_ groups). The most likely explanation for this finding is that higher PEEP, particularly at the levels used in this study (25 cmH_2_O), has marked hemodynamic effects, caused by an impairment of venous return, increase in pulmonary artery pressure, and decrease in cardiac output (Cournand et al., 1947; Mahmood & Pinsky, 2018; Soni & Williams, 2008). In turn, this hemodynamic pattern activates several homeostatic mechanisms, such as sympathetic system, glomerulo‐tubular feedback, and renin‐angiotensin‐aldosterone system, which lead to a decrease of glomerular filtration rate, with consequent alteration of sodium and chloride reabsorption (Rose & Post, 2001). Actually, in both experimental groups treated with higher PEEP (L_VENT_‐H_PEEP_ and I_VENT_‐H_PEEP_ groups), glomerular filtration rate steadily decreased, and the plasma creatinine and urea levels increased compared to lower PEEP groups (see Figure 6 Figure S3).

Along the same line of reasoning, a convenient way to quantify the kidney response to PaCO_2_ variations over a given period of time is to use the renal compensation ratio. These ratio—that is, the SID change per unit change of PaCO_2_—was significantly higher in low PEEP groups, while was close to zero—that is, absence of compensation—in the higher PEEP groups (see Figure 3). This behavior likely reflects the altered hemodynamic pattern and its consequences on renal function, as discussed above.

### 4.3 Ventilatory setting and renal function

That PEEP rather than high‐minute ventilation resulted in a change in renal function may be surprising because high‐minute ventilation has been reported to be associated with renal injury (Pannu & Mehta, 2002).

In addition, contrary to expectations the group with low‐minute ventilation and higher PEEP had a significantly higher cardiac output than the other groups. These results may be explained by the different mean airway pressure and PCO_2_ levels (see Table 1). Actually, the hyper‐ventilated group had a significantly higher mean airway pressure than the hypo‐ventilated group throughout the whole experiment (see Figure S6). This led to an increase in intrathoracic pressures, with markedly different hemodynamic patterns; indeed, central venous pressure, mean arterial pressure, and mean pulmonary arterial pressure were significantly higher in groups treated with higher PEEP during the whole experimental phase. However, impaired renal function cannot be directly attributed to a greater decrease in cardiac output: Indeed, cardiac output was significantly and markedly higher in hypo‐ventilated pigs with higher PEEP compared to pigs with higher PEEP and hypocapnia (see Figure 5d) (Kiely et al., 1996). This consistent with the effect of increased PCO_2_ on reducing systemic vascular resistance (see Figure 5c) (Walley et al., 1990). In addition, the need for vasopressors to maintain hemodynamic stability during the experimental phase must be emphasized, as these were significantly higher in pigs with higher PEEP (see Figure S7 and Table 1). Therefore, it seems plausible that higher mean airway pressure—directly and indirectly—is a key factor in determining the impairment in kidney function.

## 5 LIMITATIONS AND CONCLUSIONS

This study has several limitations: the first is related to the selection of study population, which was arbitrarily set a posteriori. In particular, due to the experimental design, we lack groups with higher PEEP associated with high‐minute ventilation and with lower PEEP associated with low‐minute ventilation; data from these groups could have strengthened the interpretation of our results. We must consider, however, that these two conditions are unusual in clinical practice: indeed, higher PEEP is usually associated with low‐minute ventilation, whereas lower PEEP values and low‐minute ventilation are not used as it unavoidably leads to progressive lung collapse (Gattinoni et al., 2017). Another possible limitation consists in the impossibility of computing the fraction of negatively charged albumin, as the estimation of dissociation constant in humans is not suitable for pigs; the computation of charged fraction of weak acid is in fact essential for the estimation of unmeasured anions, which may have developed with worsening of acute kidney injury. However, the total amount of albumin was similar among groups and similarly decreased with time. Ultimately, our analysis and conclusions are based on the interplay between two systems (i.e., plasma and the kidneys), while more interactions could represent alternative explanations for our results (e.g., third space effect, red blood cells, muscle, bones, etc). Moreover, the different amounts of fluid administered could have partially played a role in determining the observed phenomena, even if its contribution remains unquantifiable. However, consistent with our data and the literature, the kidneys seem to play the most important role in determining acid–base compensation through [SID] variations in response to altered PCO_2_.

In this study, we showed that kidney immediately and progressively over 48 h compensate the changes in PCO_2_ induced by minute ventilation adjustment, especially during hypocapnia. This response, however, can be dampened or abolished by high PEEP levels through hemodynamic impairment. Our results cannot be translated to human clinical practice, in which PEEP levels as high as used in this study (25 cmH_2_O) are rarely applied; however, our data may call attention to kidney behavior in clinical conditions in which permissive hypercapnia, when low tidal volume and higher PEEP, is applied.

## AUTHOR CONTRIBUTIONS

PT: conceived and designed the research, analyzed data, interpreted the results of the experiments, prepared figures, edited and revised the manuscript; NRV: conceived and designed the research, interpreted the results of the experiments, prepared figures, drafted manuscript, edited and revised the manuscript; FA: interpreted the results of the experiments, edited and revised the manuscript; FI: edited and revised the manuscript; RF: performed experiments, edited and revised the manuscript; BM: performed experiments, edited and revised the manuscript; CF: performed experiments, edited and revised the manuscript, approved final version of manuscript; GS: performed experiments, edited and revised the manuscript; CP: edited and revised the manuscript, approved final version of the manuscript; MO: edited and revised the manuscript, approved final version of the manuscript; QM: edited and revised the manuscript, approved final version of the manuscript; MK: edited and revised the manuscript, approved final version of the manuscript; CL: interpreted the results of the experiments, edited and revised the manuscript, approved final version of the manuscript; GL: conceived and designed the research, interpreted the results of the experiments, drafted manuscript, approved final version of the manuscript.

## FUNDING INFORMATION

None.

## ETHICS STATEMENT

The study was formerly approved by local authorities (18/2795, LAVES, Oldenburg, Niedersachsen, Germany). All experiment were performed according to the Helsinki Declaration.

## Supporting information


Data S1:

## Data Availability

The data that support the findings of this study are available from the corresponding author, L.G., upon reasonable request. Supplemental Tables and Figures: 10.6084/m9.figshare.24271090.
