# Supplementary material for: Acute renal response to changes in carbon dioxide in mechanically ventilated female pigs
Source: Physiol Rep. 2024 Sep 18;12(18):e70042. doi: 10.14814/phy2.70042 (PMC11410556; doi:10.14814/phy2.70042)

***Acute Kidney Response to Mechanical Hyper- and Hypo-ventilation***

*Pozzi T.*, Nicolardi R.V.*, Fioccola A., Fratti I., Romitti F., Busana M., Collino F., Gattarello S., J. Wieditz, Caironi P., Moerer O., Quitel M., Meissner K., Camporota L. and Gattinoni L.*

# **SUPPLEMENTAL MATERIAL**

***Additional Material and Methods***

This study was approved by local authorities (LAVES, Niedersächsisches Landesamt für Verbraucherschutz und Lebensmittelsicherheit, Projekt 18/2795). The experiments were performed in a dedicated operating room of the animal facility of Göttingen medicine university (UMG), Göttingen, Germany, between June and November 2019. The animals chosen for the experiment were healthy domestic female piglets weighing 24.2 ± 2.0 kg (about 2 months of age). The team of veterinarians administered anxiolytic premedication with azaperone 2 mg/kg and ketamine 10 mg/kg through muscular injection into the neck. Propofol (10-20 mg) was administered through a peripheral venous access (20-22 Gauge). The pigs were thoroughly washed and then placed on a special veterinary surgical bed in the operating room. The initial monitoring included oxygen saturation at the tail or the ear and 3-lead electrocardiography. After pre-oxygenation via a nose cone connected to a bag valve mask, anesthesia was induced with intravenous sufentanyl (30-50 μg) and propofol (40-80 mg) boli. Laryngoscopy was performed and endotracheal tube (6.5-7.5 mm internal diameter) was positioned with the help of a stylet. Once intubated, the animal was connected to the Avea ventilator. Mechanical ventilation was set up with the following mode before the start of the experiment: volume-controlled mode, 40% fraction of inspired oxygen, tidal volume 8-10 ml kg^-1^, respiratory rate 15-25 and PEEP 5 cmH_2_O, inspiratory-to-expiratory ratio 1:1 in order to obtain an EtCO_2_ between 35-45 mmHg. General anesthesia was maintained with propofol, sufentanyl and midazolam. Specifically, sufentanyl 2.04 ± 0.65 mcg kg^-1^hour^-1^, propofol 10.7±2 mg kg^-1^hour^-1^ and midazolam 1.06±0.39 mg kg^-1^hour^-1^. Heparin and antibiotics were also administered: heparin 1000 UI/h and amoxicillin 15 mg kg^-1^ every 24 hours.

Pigs were instrumented with the following devices:

- urinary catheter (6 Fr)
- central venous catheter (5 Fr) in the external jugular vein, ultrasound-guided
- pulmonary artery catheter (Swan-Ganz catheter) (5 Fr) through an introducer (7 Fr) in the external jugular vein, ultrasound-guided
- arterial PiCCO catheter (5 Fr) in the femoral artery, ultrasound guided.

Central temperature was continuously monitored via the Swan-Ganz catheter and hypothermia was avoided during the experiment by using warmed infusions and/or heating blankets.

Fluid management included Sterofundin ([Na^+^] = 145 mEq/L, [K^+^] = 4 mEq/L, [Ca^++^] = 5 mEq/L, [Mg^++^] = 2 mEq/L, [Cl^-^] = 127 mEq/L, [Acetate] = 24 mEq/L, [Malate] = 5 mEq/L) 4 ml kg^-1^h^-1^ via dial-a-flow and potassium and glucose when necessary to maintain normokalemia e normoglycemia.

Gelofunid 4%, Norepinephrine and Epinephrine were used to maintain a mean arterial pressure (MAP) > 60 mmHg.

Respiratory, hemodynamic and gas exchange evaluation were taken at six-hour intervals. At the conclusion of the experiment, euthanasia was administered via a pentobarbital overdose along with 40-60 mEq of potassium chloride.

Measured Variables

*Measured respiratory mechanics variables (Avea ventilator)*: fraction of inspired oxygen (FIO_2_), tidal volume, respiratory rate, inspiratory time, inspiratory-to-expiratory (I:E) ratio, inspiratory flow, minute ventilation, mean airway pressure, peak inspiratory pressure (P_peak_), airway plateau pressure during inspiratory pause (Paw_plat_), airway pressure during expiratory pause at PEEP (Paw_PEEP_), airway pressure during expiratory pause at ZEEP (Paw_ZEEP_), volume release from PEEP to ZEEP, esophageal plateau pressure during inspiratory pause (Pes_plat_), esophageal pressure during expiratory pause at PEEP (Pes_PEEP_), esophageal pressure during expiratory pause at ZEEP (Pes_ZEEP_), CO_2_ elimination (VCO_2_).

*Computed respiratory mechanics variables:*

$$Driving Pressure \left( cmH_{2}O \right)={Paw}_{PLAT}-{Paw}_{PEEP}$$

where Paw_PLAT_ and Paw_PEEP_ are airway pressures measured after 5-second-long inspiratory and expiratory hold maneuvers

$$Respiratory System Elastance(cmH_{2}O L^{-1})=\frac{{Paw}_{PLAT}-{Paw}_{PEEP}}{V_{T}}$$

where Pes_PLAT_ and Pes_PEEP_ are esophageal pressures measured after 5-second-long inspiratory and expiratory hold maneuvers and V_T_ is tidal volume in Liters.

$$Mechanical Power \left( J {min}^{-1} \right)=0.098\times V_{T}\times RR\times(Paw_{PEAK}-\frac{Driving Pressure}{2})$$

where RR is respiratory rate in bpm.

*Measured hemodynamic variables (Solar 8000, PiCCO)*: heart rate, systemic and pulmonary artery pressures (systolic, diastolic, mean), wedge and central venous pressures during expiration, thermodilution cardiac output (PiCCO).

*Derived hemodynamic variables*: systemic and pulmonary artery resistances.

*Measured gas exchange variables (GEM 3000)*: pH, PO_2_ (mmHg), PCO_2_ (mmHg), hematocrit (%), sodium (mmol/L), potassium (mmol/L), calcium (mmol/L), lactate (mmol/L)

*Fluid-related variables*: urinary output, maintenance fluids, pumps (propofol, sufentanil, midazolam, heparin, norepinephrine, epinephrine), resuscitation fluids. Fluid balance was calculated as input (maintenance, resuscitation, pumps) – output (urinary, esophageal) fluids.

***Additional Results***

**Table S1**. Baseline characteristics of the study population according to the four experimental groups. Dark grey columns refer to groups ventilated with higher PEEP (25 cmH_2_O), while light grey groups identify groups with lower PEEP (5 cmH_2_O). Bold: *p* < 0.050. L_VENT_-H_PEEP_: low minute ventilation high PEEP; I_VENT_-H_PEEP_: intermediate minute ventilation high PEEP; I_VENT_-L_PEEP_: intermediate minute ventilation low PEEP; H_VENT_-L_PEEP_: high minute ventilation low PEEP.

|  | L_VENT_-H_PEEP_  17% (7) | I_VENT_-H_PEEP_  17% (7) | I_VENT_-L_PEEP_  24% (10) | H_VENT_-L_PEEP_  42% (17) | *p* |
| --- | --- | --- | --- | --- | --- |
| Weight, *kg* | 24 [23 – 25] | 26 [23 – 26] | 25 [24 – 26] | 24 [23 – 26] | *0.401* |
| Temperature, *°C* | 35.6 [35.4 - 36.1] | 35.8 [35.5 - 36] | 35.4 [35 - 36.2] | 35.6 [35 - 36.4] | *0.984* |
| Tidal volume, *mL* | 230 [216 - 233] | 234 [212 - 246] | 242 [217 - 261] | 234 [214 - 246] | *0.417* |
| Tidal volume per kg, *mL kg^-1^* | 9 [9 - 10] | 9 [9 - 10] | 9 [9 - 10] | 10 [9 - 11] | *0.418* |
| Respiratory rate, *bpm* | 20 [19 - 22.5] | 20 [18 - 21] | 20 [20 - 22] | 20 [17 - 22] | *0.750* |
| Minute ventilation, *mL min^-1^* | 4.3 [4.2 - 4.4] | 4.9 [4.2 - 5.2] | 4.8 [3.9 - 5.3] | 4.4 [3.8 - 4.9] | *0.746* |
| Minute ventilation per kg, *mL min^-1^ kg^-1^* | 0.19 [0.16 – 0.20] | 0.18 [0.17 - 0.19] | 0.19 [0.17 - 0.21] | 0.18 [0.17 - 0.21] | *0.855* |
| PEEP, *cmH_2_O* | 5 | 5 | 5 | 5 | *-* |
| FiO_2_ | 0.40 | 0.40 | 0.40 | 0.40 | *-* |
| Peak pressure, *cmH_2_O* | 17.3 [17.1 - 17.9] | 16.1 [15.0 - 17.1] | 16.1 [15.0 - 17.1] | 16.6 [15.6 - 17.7] | *0.351* |
| Plateau pressure, *cmH_2_O* | 14.6 [14.1 - 15.0] | 14.1 [13.3 - 14.5] | 13.8 [13.1 - 14.9] | 14.4 [13.8 - 14.9] | *0.191* |
| Driving pressure, *cmH_2_O* | 9.5 [8.8 - 10.3] | 8.7 [8.0 - 9.1] | 8.9 [8.0 - 10.2] | 9.0 [8.7 - 9.7] | *0.332* |
| Mean airway pressure, *cmH_2_O* | 9 [9 – 10] | 9 [9 – 10] | 9 [8 – 9] | 9 [9 – 10] | *0.285* |
| Respiratory system elastance, *cmH_2_O L^-1^* | 43.8 [40.9 – 44.3] | 35.3 [32.8-44.5] | 36.4 [34.1 – 38.2] | 38.9 [35.9 – 42.0] | *0.091* |
| Mechanical power, *J min^-1^* | 5.1 [4.9 – 5.2] | 5.1 [4.4 – 5.6] | 5.3 [4.4 – 6.3] | 5.3 [4.4 – 6.1] | *0.893* |
| Arterial pH | 7.49 [7.48 - 7.54] | 7.517.44 - 7.54] | 7.52 [7.50 - 7.55] | 7.52 [7.46 - 7.56] | *0.597* |
| PaCO_2,_ *mmHg* | 43.5 [41.5 - 47.5] | 44 [39 - 44] | 43 [41.2 - 44] | 43 [42 - 46] | *0.920* |
| VCO_2_, *mL min^-1^* | 128 [126 – 137] | 136 [117 – 141] | 130 [109 – 138] | 129 [114 – 158] | *0.696* |
| HCO_3_^-^, *mMol L^-1^* | 33.5 [32.2 - 34.7] | 34.1 [33.2 - 34.8] | 34.3 [34.3 - 35.5] | 35.4 [33.8 – 37.0] | *0.233* |
| PaO_2,_ *mmHg* | 221 [210 – 237] | 225 [218 – 233] | 232 [228 – 243] | 226 [218 – 235] | *0.193* |
| PaO_2_/FiO_2_, *mmHg* | 547 [539 – 570] | 551 [545 – 580] | 562 [550 – 559] | 550 [534 – 574] | *0.572* |
| Plasmatic [Na^+^], *mEq L^-1^* | 144 [142 - 145] | 145[144 – 146] | 143[141 - 145] | 145 [144 - 147] | *0.125* |
| Plasmatic [K^+^], *mEq L^-1^* | 4 [3.95 - 4.15] | 4 [3.9 - 4.5] | 3.95 [3.82 - 4.3] | 4 [3.9 - 4.5] | *0.354* |
| Plasmatic [Cl^-^], *mEq L^-1^* | 105 [104 - 106] | 107 [104 - 108] | 104 [102 - 104] | 104 [103 - 107] | *0.353* |
| Plasmatic [Lac], *mMol L^-1^* | 0.5[0.4 – 0.6] | 0.7 [0.3 – 1.4] | 0.5 [05 - 0.6] | 0.5 [0.4 – 0.7] | *0.688* |
| Plasmatic [SID], *mEq L^-1^* | 43.6 [41.7 - 43.9] | 42.7 [41.4 - 43.9] | 43.2 [41.8 - 44.7] | 46.1 [44.4 - 46.5] | *0.093* |
| Central venous pressure, *cmH_2_O* | 7 [5 – 7] | 6 [5 – 7] | 8 [6 – 10] | 7 [4 – 9] | *0.418* |
| Mean pulmonary arterial pressure, *mmHg* | 19 [19 - 21] | 21 [19 - 21] | 21 [19 - 23] | 20 [18 - 26] | *0.940* |
| Systemic vascular resistance, *dyn sec cm^-5^* | 1210 [1176 – 1341] | 1273 [1158 – 1357] | 1393 [943 – 1514] | 1234 [1166 – 1493] | *0.616* |
| Heart rate, *bpm* | 102 [87 - 111] | 81 [81 - 106] | 103 [93 - 112] | 101 [83 - 112] | *0.720* |
| Stroke volume, *mL* | 42 [37 – 43] | 42 [42 – 45] | 41 [40 – 46] | 39 [35 – 46] | *0.981* |
| Cardiac output, *L min^-1^* | 4.1 [3.6 - 4.35] | 4.25 [3.78 - 4.80] | 4.2 [3.82 - 4.88] | 3.8 [3.5 - 4.6] | *0.859* |
| Creatinine, *mg dL^-1^* | 1.0 [0.8 – 1.2] | 1.1 [1.0 – 1.2] | 1.1 [1.0 – 1.2] | 1.1 [1.0 – 1.1] | *0.402* |
| Blood urea nitrogen, *mg dL^-1^* | 8 [7.5 – 10.5] | 7 [6.5 – 9] | 8 [7 – 9.5] | 8 [7 – 10] | *0.406* |
| Urinary [Na^+^], *mEq L^-1^* | 43 [34 – 56] | 52 [27 – 69] | 41 [32 – 45] | 46 [22 – 67] | *0.756* |
| Urinary [K^+^], *mEq L^-1^* | 63 [50-105] | 113 [105 – 132] | 81 [63 – 96] | 68 [44 – 107] | ***0.033*** |
| Urinary [Cl^-^], *mEq L^-1^* | 44 [38 – 71] | 49 [46 - 87] | 60 [34 – 84] | 67 [48 – 88] | *0.845* |
| Urinary [SID], *mEq L^-1^* | 42 [37 – 96] | 76 [71 – 120] | 62 [38 – 95] | 44 [27 – 69] | *0.230* |

**Table S2**. Annotations as in Figure S1. Time course of all acid-base, hemodynamic, respiratory and gas exchange variables measured or computed. Dark grey columns refer to groups ventilated with higher PEEP (25 cmH_2_O), while light grey groups identify groups with lower PEEP (5 cmH_2_O). Bold: *p* < 0.050.

|  | L_VENT_-H_PEEP_  17% (7) | I_VENT_-H_PEEP_  17% (7) | I_VENT_-L_PEEP_  24% (10) | H_VENT_-L_PEEP_  42% (17) | *p_GROUP_* | *p_TIME_* | *p_INTER_* |
| --- | --- | --- | --- | --- | --- | --- | --- |
| Temperature, *°C*  *After 0.5 hours*  *After 6 hours*  *After 48 hours* | 36.4 [35.8 – 37.1]  39.0 [38.1 – 39.2]  38.5 [38.2 – 38.7] | 36.1 [35.8 – 36.4]  37.6 [37.3 – 37.7]  37.4 [36.7 – 40.1] | 35.6 [35.1 – 36.1]  38.6 [37.8 – 39.9]  38.2 [37.7 – 38.9] | 36.1 [35.8 – 36.9]  38.3 [37.7 – 39.4]  36.9 [36.6 – 38.2] | *0.449* | ***0.001*** | *0.058* |
| Tidal volume, *mL*  *After 0.5 hours* | 320 [310 – 330] | 330 [300 – 350] | 740 [330 – 860] | 360 [320 – 690] | *0.130* | *-* | *-* |
| Tidal volume per kg, *mL kg^-1^*  *After 0.5 hours* | 13.8 [12.8 – 14.3] | 13.6 [12.9 – 14.9] | 30.1 [14.1 – 34.4] | 14.1 [13.1 – 30.9] | *0.379* | *-* | *-* |
| Respiratory rate, *bpm*  *After 0.5 hours* | 11 [11 – 12] | 22 [20 – 22]* | 8 [6 – 32] | 40 [16 – 40]*^§^ | ***<0.001*** | *-* | *-* |
| Minute ventilation, *mL min^-1^*  *After 0.5 hours* | 3.7 [3.3 – 3.7] | 6.8 [6.6 – 7.7]* | 6.1 [5.4 – 7.7]* | 12.6 [10.9 – 14.3]*°^§^ | ***<0.001*** | *-* | *-* |
| Minute ventilation per kg, *mL min^-1^ kg^-1^*  *After 0.5 hours* | 0.15 [0.14 – 0.15] | 0.30 [0.28 - 0.31]* | 0.25 [0.22 - 0.35]* | 0.50 [0.48 - 0.56]*°^§^ | ***<0.001*** | *-* | *-* |
| PEEP, *cmH_2_O*  *After 0.5 hours* | 25 | 25 | 5 | 5 | *-* | *-* | *-* |
| FiO_2_  *After 0.5 hours* | 0.40 | 0.40 | 0.40 | 0.40 | *-* | *-* | *-* |
| Peak pressure, *cmH_2_O*  *After 0.5 hours*  *After 6 hours*  *After 48 hours* | 51 [50 – 52]  46 [45 – 48]  46 [44 – 47] | 58 [53 – 63]  54 [49 – 58]  50 [49 – 51] | 33 [24 – 42]  35 [23 – 40]  37 [30 – 46] | 48 [26 – 35]  30 [23 – 32]  35 [30 – 42] | ***<0.001*** | *0.389* | ***0.001*** |
| Plateau pressure, *cmH_2_O*  *After 0.5 hours*  *After 6 hours*  *After 48 hours* | 48 [46 - 49]  42 [42 – 45]  42 [41 – 43] | 52 [50 – 59]  50 [45 – 53]  45 [43 – 47] | 29 [20 – 33]  29 [19 – 32]  29 [26 – 35] | 23 [20 – 28]  25 [18 – 27]  27 [24 – 34] | ***<0.001*** | *0.171* | ***<0.001*** |
| Driving pressure, *cmH_2_O*  *After 0.5 hours*  *After 6 hours*  *After 48 hours* | 24 [23 – 26]  18 [18 – 21]  18 [17 – 19] | 29 [27 – 34]  26 [24 – 27]  20 [19 – 22] | 24 [13 – 28]  24 [14 – 28]  23 [20 – 30] | 12 [10 – 22]  14 [10 – 20]  18 [15 – 26] | ***0.016*** | *0.058* | ***<0.001*** |
| Mean airway pressure, *cmH_2_O*  *After 0.5 hours*  *After 6 hours*  *After 48 hours* | 30 [29 – 31]  29 [28 – 31]  28 [28 – 29] | 36 [36 – 39]*  33 [29 – 36]*  33 [32 – 35]* | 11 [10 – 11]*°  11 [9 – 12]*°  11 [10 – 12]*° | 15 [13 – 16]*°^§^  15 [13 – 16]*°^§^  17 [16 – 20]*°^§^ | ***<0.001*** | *0.070* | ***<0.001*** |
| Respiratory system elastance, *cmH_2_O L^-1^*  *After 0.5 hours*  *After 6 hours*  *After 48 hours* | 74.4 [72.9 – 79.4]  58.4 [53.2 – 61.3]  54.9 [49.6 – 58.6] | 88.1 [77.0 – 105.5]  75.3 [66.1 – 80.9]  74.0 [69.3 – 74.6] | 34.9 [32.5 – 36.2]  35.1 [33.1 – 39.9]  36.7 [32.4 – 43.2] | 33.9 [29.5 – 36.7]  35.7 [33.9 – 40.2]  47.1 [41.5 – 64.5] | ***<0.001*** | *0.093* | ***<0.001*** |
| Mechanical power, *J min^-1^*  *After 0.5 hours*  *After 6 hours*  *After 48 hours* | 13.7 [13.2 – 14.2]  13.2 [12.5 – 13.3]  13.1 [12.5 – 13.8] | 28.9 [27.8 – 31.5]  28.4 [26.7 – 30.1]  24.1 [23.0 – 25.7] | 14.1 [12.9 – 14.2]  13.4 [12.5 – 14.4]  14.9 [13.2 – 15.9] | 29.8 [25.5 – 30.5]  27.9 [23.0 – 32.4]  33.6 [25.6 – 39.7] | ***<0.001*** | *0.406* | ***0.002*** |
| Arterial pH  *After 0.5 hours*  *After 6 hours*  *After 48 hours* | 7.38 [7.33 – 7.42]  7.27 [7.25 – 7.31]  7.22 [7.22 – 7.26] | 7.58 [7.54 – 7.62]*  7.58 [7.57 – 7.71]*  7.47 [7.43 – 7.54]* | 7.66 [7.64 – 7.68]*°  7.67 [7.63 – 7.69]*  7.53 [7.51 – 7.55]* | 7.80 [7.76 – 7.80]*°^§^  7.80 [7.78 – 7.80]*°^§^  7.60 [7.56 – 7.63]*°^§^ | ***<0.001*** | ***<0.001*** | ***<0.001*** |
| PaCO_2,_ *mmHg*  *After 0.5 hours*  *After 6 hours*  *After 48 hours* | 51 [50 – 56]  60 [55 – 65]  60 [55 – 69] | 31 [30 – 35]*  30 [28 – 33]*  35 [33 – 36]* | 29 [27 – 30]*  26 [24 – 30]*  24 [21 – 28]* | 15 [15 – 20]*°^§^  14 [11 – 16]*°^§^  13 [12 – 17]*°^§^ | ***<0.001*** | *0.615* | ***<0.001*** |
| VCO_2,_ *mL min^-1^*  *After 0.5 hours*  *After 6 hours*  *After 48 hours* | 149 [131 – 163]  195 [174 – 204]  170 [155 – 191] | 173 [164 – 184]  151 [143 – 166]  140 [124 – 154] | 146 [136 – 156]  152 [151 – 169]  126 [124 – 131]* | 166 [149 – 192]  156 [141 – 179]  131 [126 – 138]* | *0.087* | ***<0.001*** | ***<0.001*** |
| [HCO_3_^-^], *mmol L^-1^*  *After 0.5 hours*  *After 6 hours*  *After 48 hours* | 30.6 [27.8 – 32.4]  27.3 [26.9 – 30.2]  24.2 [22.2 – 28.7] | 30.1 [27.8 – 32.4]  28.1 [26.7 – 29.1]  24.0 [22.0 – 25.3] | 31.5 [30.2 – 32.7]  30.4 [28.1 – 31.7]  20.6 [17.5 – 22.4]* | 33.8 [32.8 – 35.4]°  31.2 [25.3 – 35.1]  13.0 [11.6 – 16.7]*°^§^ | ***0.038*** | ***<0.001*** | ***<0.001*** |
| PaO_2,_ *mmHg*  *After 0.5 hours*  *After 6 hours*  *After 48 hours* | 218 [203 – 220]  202 [175 – 208]  196 [180 – 200] | 254 [249 – 254]  244 [227 – 249]  237 [234 – 244] | 246 [238 – 255]  211 [207 – 222]  221 [211 – 234] | 254 [244 – 261]  238 [231 – 248]  239 [238 – 255] | ***<0.001*** | ***<0.001*** | *0.174* |
| PaO_2_/FiO_2_, *mmHg*  *After 0.5 hours*  *After 6 hours*  *After 48 hours* | 555 [508 – 574]  460 [419 – 472]  445 [410 – 483] | 618 [614 – 623]*  573 [565 – 605]*  562 [539 - 570]* | 605 [579 – 622]*  535 [508 – 559]*  540 [527 – 571]* | 625 [600 – 639]*  595 [575 – 605]*^§^  588 [569 – 621]*^§^ | ***<0.001*** | ***<0.001*** | ***0.002*** |
| Plasmatic [Na^+^], *mEq L^-1^*  *Baseline*  *After 6 hours*  *After 48 hours* | 144 [142 – 145]  147 [147 – 148]  148 [146 – 148] | 145 [144 – 145]  147 [146 – 147]  148 [146 – 149] | 143 [141 – 145]  146 [142 – 148]  146 [144 – 147] | 145 [144 – 147]  146 [143 – 149]  148 [146 – 150] | *0.191* | ***<0.001*** | *0.424* |
| Plasmatic [K^+^], *mEq L^-1^*  *Baseline*  *After 6 hours*  *After 48 hours* | 4.0 [3.9 – 4.2]  5.1 [5.0 – 5.2]  7.1 [5.9 – 7.1] | 4.0 [3.9 – 4.4]  4.6 [4.3 – 5.0]  5.5 [4.6 – 5.7] | 3.9 [3.8 – 4.3]  4.6 [4.4 – 5.2]  3.9 [3.7 – 4.0]*° | 4.0 [3.9 – 4.5]  4.1 [4.0 – 4.5]*  3.8 [3.5 – 4.0]*° | ***<0.001*** | ***<0.001*** | ***<0.001*** |
| Plasmatic [Cl^-^], *mEq L^-1^*  *Baseline*  *After 6 hours*  *After 48 hours* | 105 [104 - 106]  110 [108 – 112]  111 [108 – 112] | 107 [104 – 107]  110 [109 – 113]  113 [113 – 116] | 104 [101 – 104]  110 [106 – 111]  119 [117 – 120]*° | 104 [103 - 107]  112 [111 – 115]  124 [123 – 126]*°^§^ | ***<0.001*** | ***<0.001*** | ***<0.001*** |
| Plasmatic [Lac], *mEq L^-1^*  *Baseline*  *After 6 hours*  *After 48 hours* | 0.5 [0.4 – 0.6]  2.1 [0.7 – 2.6]  1.0 [0.6 – 1.0] | 0.7 [0.3 – 1.4]  1.4 [1.1. – 1.8]  1.0 [1.0 – 1.1] | 0.5 [0.5 - 0.6]  0.9 [0.7 – 0.9]  0.5 [0.4 – 0.7] | 0.5 [0.4 – 0.7]  1.6 [1.2 – 2.0]  1.0 [0.9 – 1.3] | ***0.007*** | ***<0.001*** | *0.067* |
| Plasmatic [SID], *mEq L^-1^*  *Baseline*  *After 6 hours*  *After 48 hours* | 43.6 [41.7 - 43.9]  42.5 [40.3 – 43.1]  43.7 [42.0 – 45.4] | 42.7 [41.4 - 43.9]  40.5 [39.4 – 42.7]  39.2 [38.8 – 40.6] | 43.2 [41.8 - 44.7]  40.8 [39.8 – 41.6]  31.8 [30.1 – 32.2]*° | 46.1 [44.4 - 46.5]  36.6 [35.7 – 38.2]*°^§^  27.4 [25.2 – 29.6]*°^§^ | ***<0.001*** | ***<0.001*** | ***<0.001*** |
| Central venous pressure, *mmHg*  *After 0.5 hours*  *After 6 hours*  *After 48 hours* | 11 [11 – 15]  11 [11 – 14]  15 [15 – 16] | 12 [8 – 17]  14 [8 – 18]  17 [16 -18] | 7 [5 – 10]  9 [5 – 11]  9 [8 – 11] | 8 [6 – 11]  6 [5 – 8]  12 [8 – 14] | ***<0.001*** | ***<0.001*** | *0.401* |
| Mean pulmonary arterial pressure, *mmHg*  *After 0.5 hours*  *After 6 hours*  *After 48 hours* | 35 [32 – 36]  37 [34 – 38]  36 [34 – 38] | 37 [36 – 38]  39 [38 – 40]  38 [37 – 40] | 21 [17 – 21]  20 [19 – 21]  24 [17 – 29] | 23 [19 – 26]  20 [17 – 23]  27 [20 – 32] | ***<0.001*** | *0.077* | *0.131* |
| Systemic vascular resistance, *dyn sec cm^-5^*  *After 0.5 hours*  *After 6 hours*  *After 48 hours* | 1066 [943 – 1217]  1004 [932 – 1056]  595 [540 – 827] | 1040 [983 – 1148]  1287 [1204 – 1772]  1146 [982 – 1156] | 1434 [1044 – 1469]  1737 [1558 – 2026]*  1225 [1059 – 1500] | 1172 [859 – 1296]  1494 [1179 – 1736]  1251 [1202 – 1678]* | *0.123* | ***0.007*** | ***0.026*** |
| Heart rate, *bpm*  *After 0.5 hours*  *After 6 hours*  *After 48 hours* | 125 [121 - 149]  138 [113 – 153]  125 [108 – 139] | 148 [138 - 175]  109 [89 – 120]  96 [85 – 110] | 101 [87 - 123]  96 [85 – 109]*  101 [87 – 123] | 139 [123 – 154]^§^  121 [109 – 136]^§^  88 [74 – 116] | ***0.006*** | ***<0.001*** | ***0.016*** |
| Stroke volume, *mL*  *After 0.5 hours*  *After 6 hours*  *After 48 hours* | 36 [25 – 38]  31 [24 – 35]  52 [45 – 59] | 29 [24 – 38]  23 [21 – 33]  36 [29 – 36] | 40 [34 – 45]  33 [26 – 35]  32 [27 – 39] | 38 [30 – 43]  30 [25 – 33]  32 [28 – 41] | *0.167* | ***<0.001*** | *0.054* |
| Cardiac output, *L min^-1^*  *After 0.5 hours*  *After 6 hours*  *After 48 hours* | 4.6 [3.6 – 4.7]  3.9 [3.6 – 4.4]  7.5 [6.3 – 7.7] | 4.7 [4.1 – 4.9]  3.4 [2.6 – 3.6]  3.5 [3.1 – 4.7]* | 4.8 [3.5 – 5.0]  3.0 [2.8 – 3.2]  3.1 [2.8 – 3.4]*° | 4.8 [4.4 – 5.8]  3.8 [2.9 – 4.4]  2.9 [2.7 – 3.6]*°^§^ | ***0.004*** | ***0.001*** | ***<0.001*** |
| Cumulative infusion, *L*  *After 0.5 hours*  *After 6 hours*  *After 48 hours* | 0.9 [0.8 – 1.1]  4.8 [4.5 – 5.1]  24.8 [22.7 – 25.5] | 0.7 [0.6 – 1.3]  4.6 [3.8 – 4.6]  18.4 [18.0 – 18.8]* | 0.7 [0.7 – 1.1]  2.5 [2.3 – 2.6]*°  15.1 [14.2 – 16.0]*° | 0.8 [0.6 – 1.3]  3.7 [3.2 – 4.2]*^§^  16.5 [16.0 – 19.3]* | ***<0.001*** | ***<0.001*** | ***<0.001*** |
| Cumulative urinary output, *mL*  *After 0.5 hours*  *After 6 hours*  *After 48 hours* | 0.1 [0.1 – 0.3]  0.3 [0.2 – 0.6]  0.7 [0.3 – 1.3] | 0.1 [0.1 – 0.2]  0.4 [0.3 – 0.6]  1.1 [0.9 – 1.8] | 0.2 [0.1 – 0.3]  0.8 [0.6 – 1.0]  2.7 [2.0 – 3.5]*° | 0.2 [0.1 – 0.5]  0.7 [0.5 – 1.3]  3.0 [2.2 – 3.7]*° | ***<0.001*** | ***<0.001*** | ***<0.001*** |
| Fluid balance, *mL*  *After 0.5 hours*  *After 6 hours*  *After 48 hours* | 1.8 [1.5 – 2.0]  3.8 [3.7 – 4.2]  12.9 [11.8 – 13.8] | 1.6 [1.4 – 2.0]  3.6 [2.8 – 3.9]  10.9 [10.2 – 11.0] | 1.1 [0.8 – 1.3]  1.8 [1.5 – 2.0]*°  8.2 [7.6 – 8.7]*° | 1.5 [1.2 – 2.1]  2.9 [2.2 – 3.3]*^§^  8.9 [8.0 – 9.8]* | ***<0.001*** | ***<0.001*** | ***<0.001*** |
| Creatinine, *mg dL^-1^*  *Baseline*  *After 6 hours*  *After 48 hours* | 1.0 [0.8 – 1.2]  1.3 [1.2 – 1.4]  3.9 [3.6 – 5.2] | 1.1 [1.0 – 1.2]  1.4 [1.3 – 1.5]  4.7 [3.9 – 4.9] | 1.1 [1.0 – 1.2]  1.4 [1.4 – 1.5]  1.5 [1.3 – 1.8]*° | 1.1 [1.0 – 1.1]  1.4 [1.3 – 1.6]  1.7 [1.3 – 2.1]*° | ***<0.001*** | ***<0.001*** | ***<0.001*** |
| Blood urea nitrogen, *mg dL^-1^*  *Baseline*  *After 6 hours*  *After 48 hours* | 8 [7 – 11]  15 [11 – 16]  47 [44 – 57] | 7 [6 – 9]  10 [9 – 12]  56 [45 – 57] | 8 [7 – 9.5]  11 [10 – 11]  19 [17 – 20]*° | 8 [7 – 10]  11 [10 – 12]  24 [21 – 27]*° | ***<0.001*** | ***<0.001*** | ***<0.001*** |
| Urinary [Na^+^], *mEq L^-1^*  *Baseline*  *After 6 hours*  *After 48 hours* | 43 [34 – 56]  47 [23 – 60]  20 [20 – 23] | 52 [27 – 69]  42 [35 – 62]  21 [20 – 22] | 41 [32 – 45]  37 [31 – 53]  87 [43 – 107]*° | 46 [22 – 67]  85 [50 – 106]^§^  65 [24 – 77] | ***0.020*** | *0.520* | ***0.006*** |
| Urinary [K^+^], *mEq L^-1^*  *Baseline*  *After 6 hours*  *After 48 hours* | 63 [50 - 105]  59 [43 – 73]  71 [66 – 82] | 113 [105 – 132]  50 [44 – 92]  102 [130 – 142] | 81 [63 – 96]  45 [31 – 80]  91 [66 – 122] | 68 [44 – 107]  50 [43 – 102]  133 [84 – 161] | *0.156* | ***0.002*** | *0.051* |
| Urinary [Cl^-^], *mEq L^-1^*  *Baseline*  *After 6 hours*  *After 48 hours* | 44 [38 – 71]  21 [20 – 37]  46 [20 – 75] | 49 [46 - 87]  22 [20 – 39]  25 [21 – 30] | 60 [34 – 84]  23 [20 – 61]  94 [40 – 138] | 67 [48 – 88]  21 [20 – 34]  70 [46 – 89] | *0.152* | ***<0.001*** | *0.139* |
| Urinary [SID], *mEq L^-1^*  *Baseline*  *After 6 hours*  *After 48 hours* | 42 [37 – 96]  53 [37 – 74]  49 [40 – 59] | 76 [71 – 120]  77 [64 – 129]  125 [111 – 132] | 62 [38 – 95]  54 [42 – 97]  69 [46 – 132] | 44 [27 – 69]  114 [64 – 145]  113 [61 – 146]* | *0.162* | *0.082* | ***0.005*** |

**Figure S1**. Symbols and annotations: L_VENT_-H_PEEP_: low minute ventilation high PEEP (blue triangles); I_VENT_-H_PEEP_: intermediate minute ventilation high PEEP (green triangles); I_VENT_-L_PEEP_: intermediate minute ventilation low PEEP (green circles); H_VENT_-L_PEEP_: high minute ventilation low PEEP (red circles). The dashed and the solid lines connects groups treated with 5 or 25 cmH_2_O of PEEP, respectively. Note that, for the sake of clarity, the intervals between measurements at baseline and 0.5 hours has been arbitrarily equated to an interval of 6 hours.

Time course of cumulative fluid intake, urinary output and fluid balance in the four experimental groups.


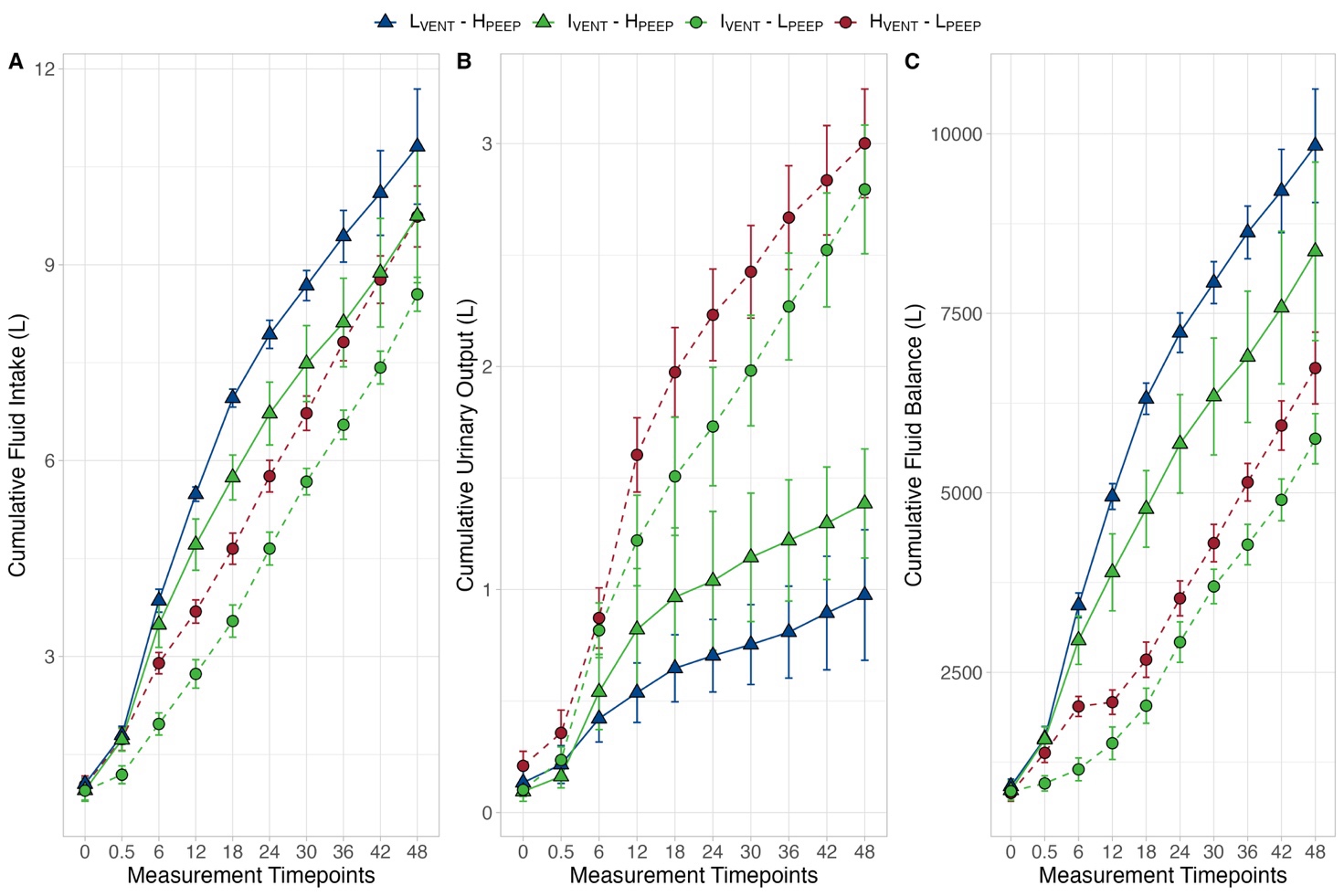


**Figure S2**. Symbols and annotation as in Figure 1. Differences among groups within time were assessed by a two-ways repeated measures ANOVA.

Time course of urinary SID (Panel A), urinary sodium (Panel B), urinary potassium (Panel C) and urinary chloride (Panel D) in the four experimental groups.


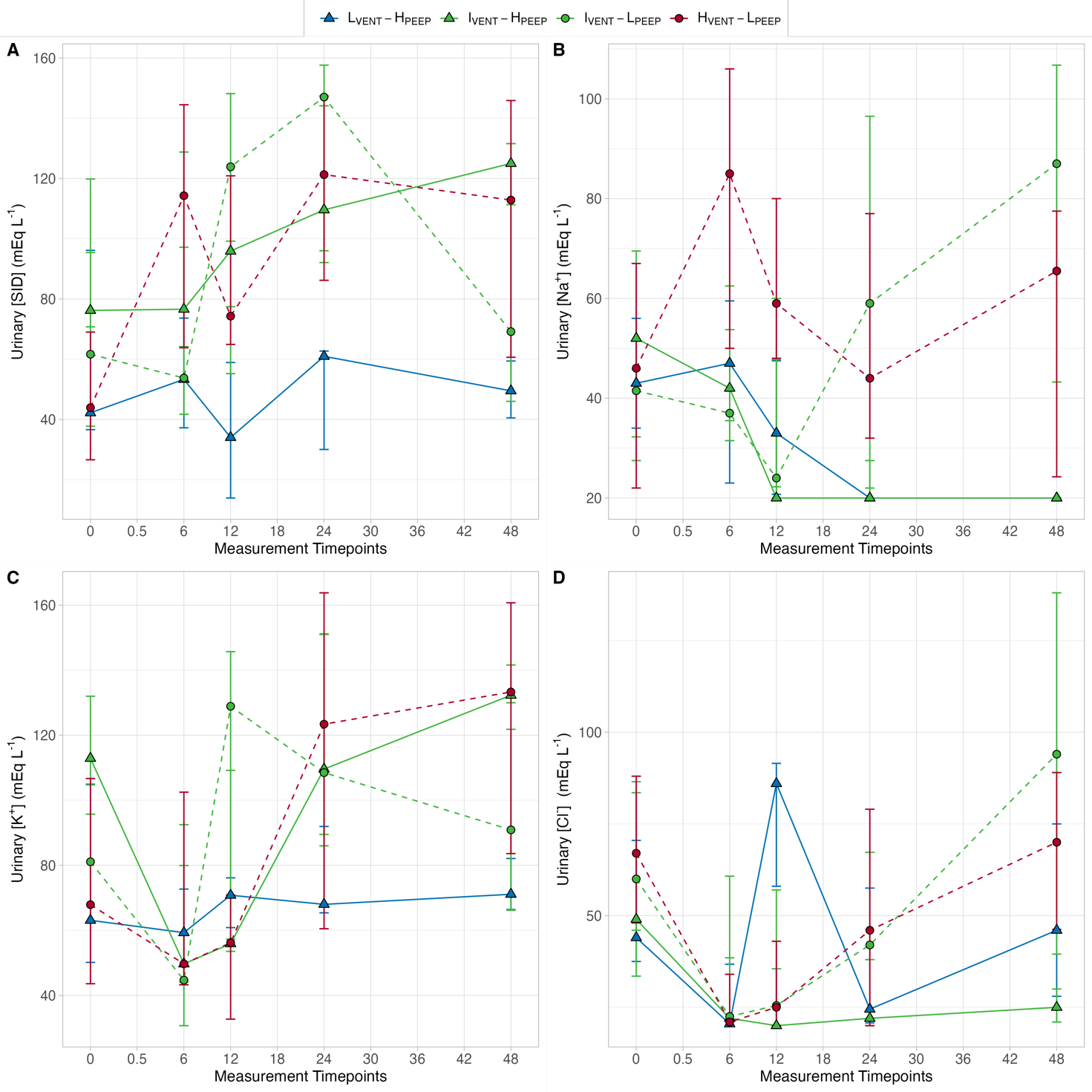


**Figure S3**. Symbols and annotation as in Figure 1. Differences among groups within time were assessed by a two-ways repeated measures ANOVA.

Time course of plasmatic blood urea nitrogen (BUN) in the four experimental groups.


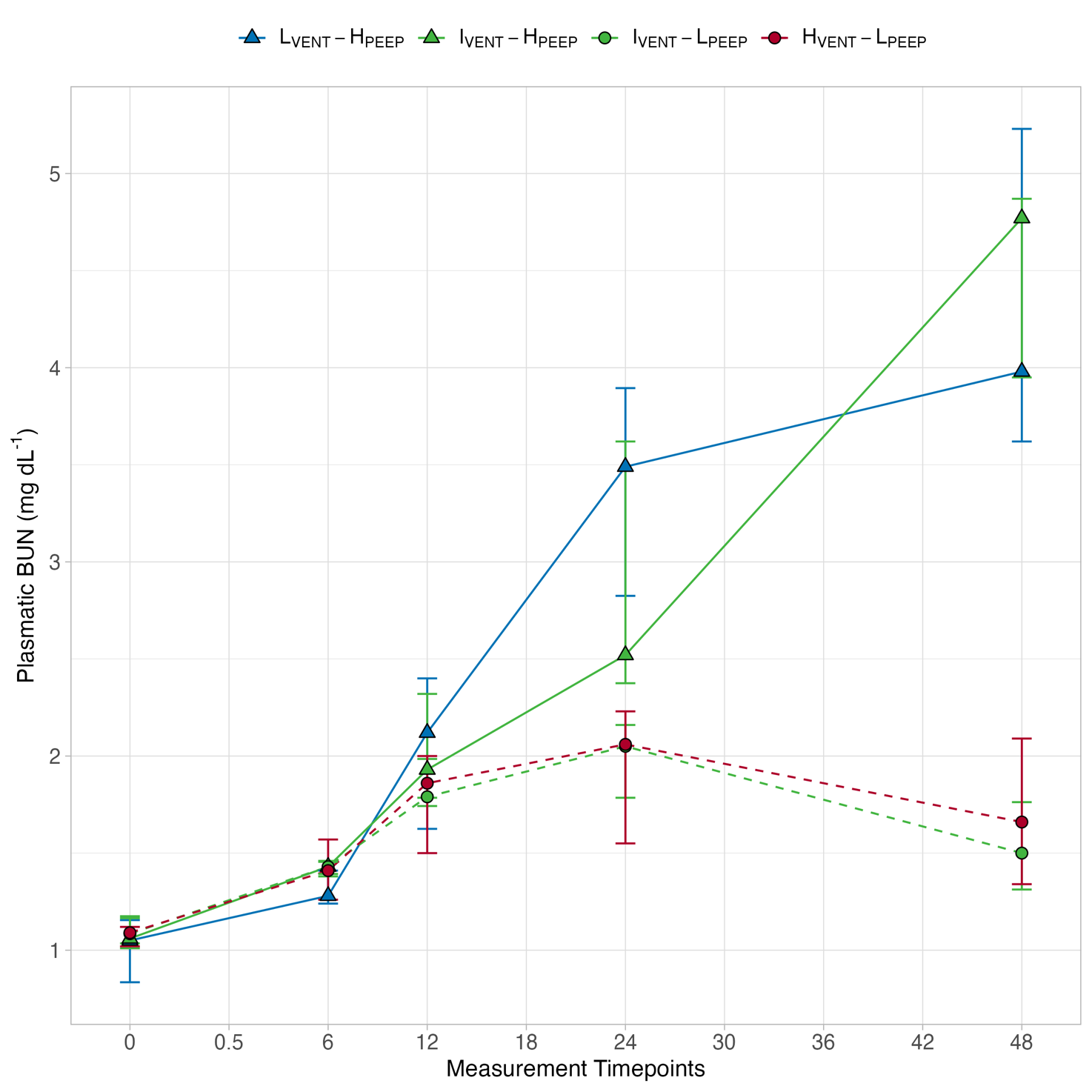


**Figure S4**. Symbols and annotations: L_VENT_-H_PEEP_: low minute ventilation high PEEP (blue); I_VENT_-H_PEEP_: intermediate minute ventilation high PEEP (green); I_VENT_-L_PEEP_: intermediate minute ventilation low PEEP (green); H_VENT_-L_PEEP_: high minute ventilation low PEEP (red).

Selection procedure of the three clusters according to minute ventilation and arterial carbon dioxide partial pressure (PaCO_2_).


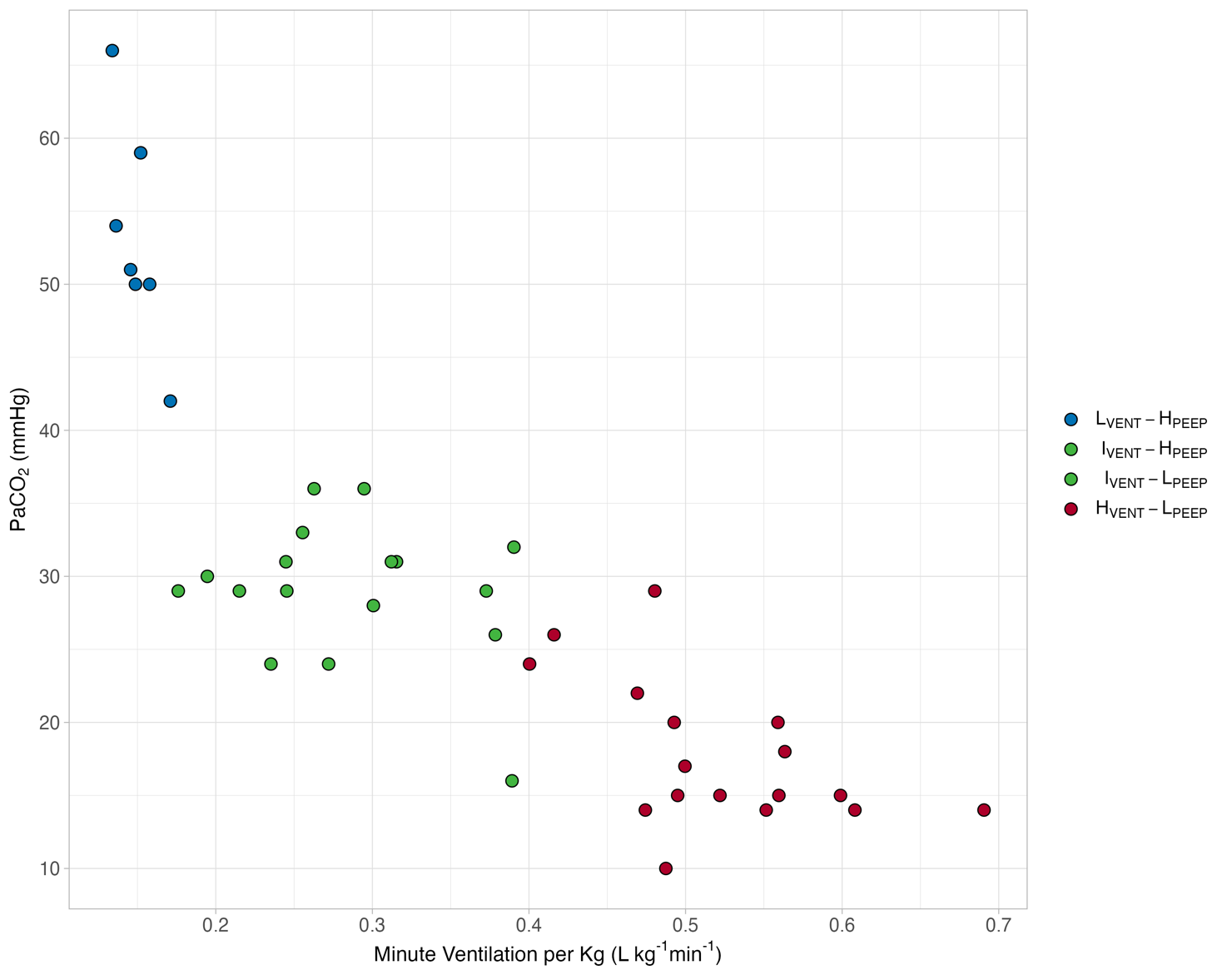


**Figure S5**. Symbols and annotation as in Figure 1. Differences among groups within time were assessed by a two-ways repeated measures ANOVA.

Time course of expired minute carbon dioxide (VCO_2_) in the four experimental groups.


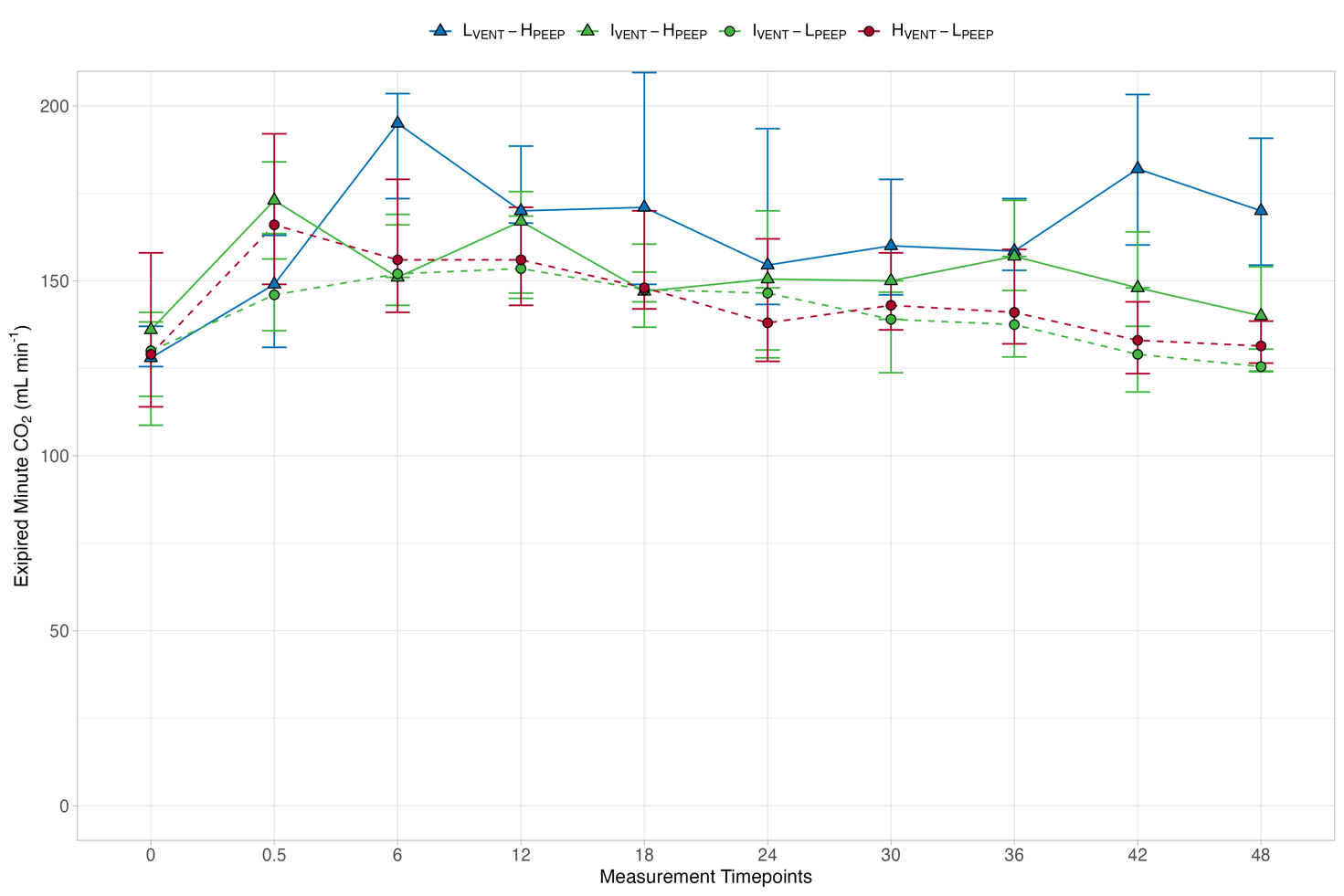


**Figure S6**. Symbols and annotation as in Figure 1. Differences among groups within time were assessed by a two-ways repeated measures ANOVA.

Time course of mean airway pressure in the four experimental groups.


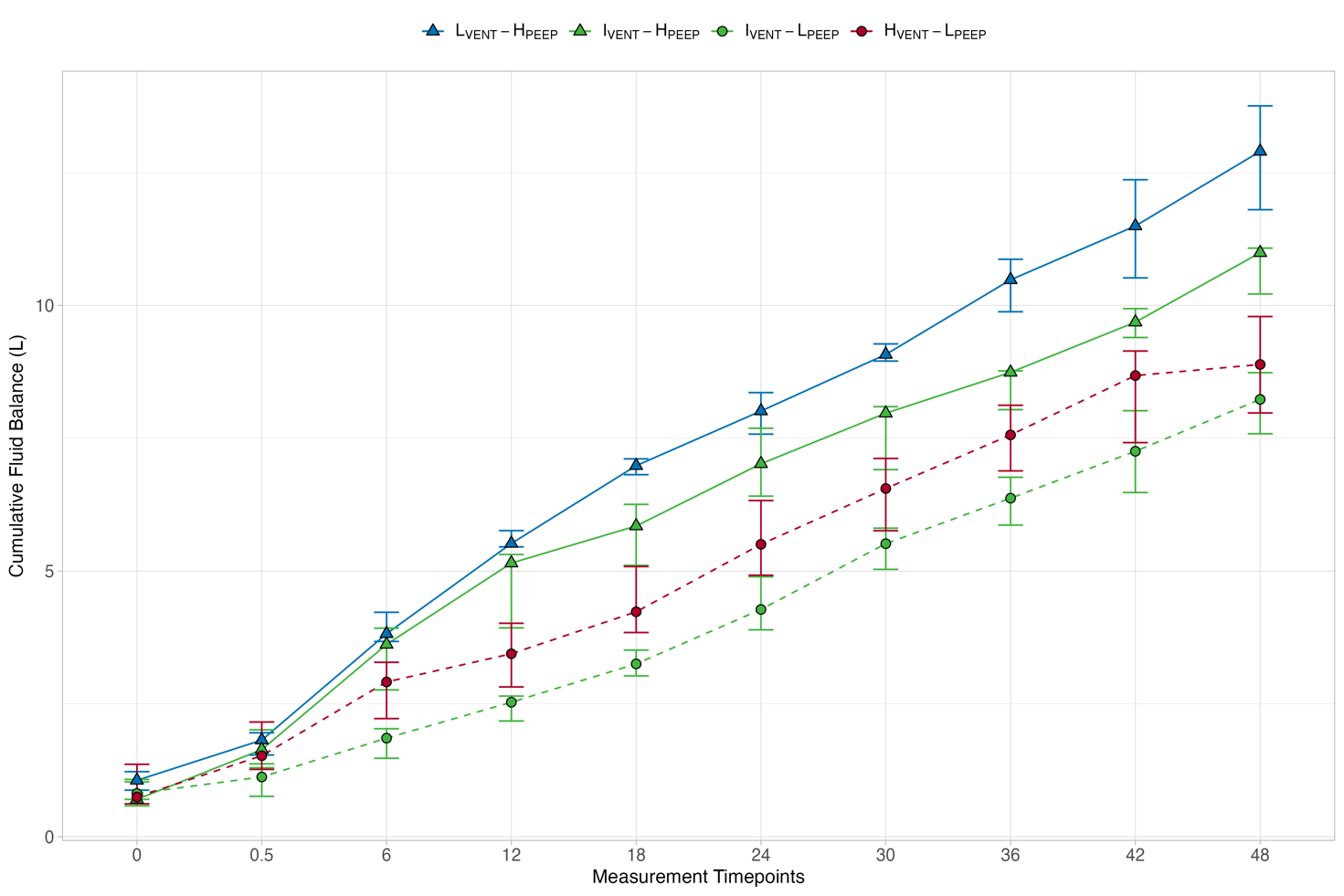


**Figure S7**. Symbols and annotation as in Figure 1. Differences among groups within time were assessed by a two-ways repeated measures ANOVA.

Time course of cumulative norepinephrine infusion in the four experimental groups.


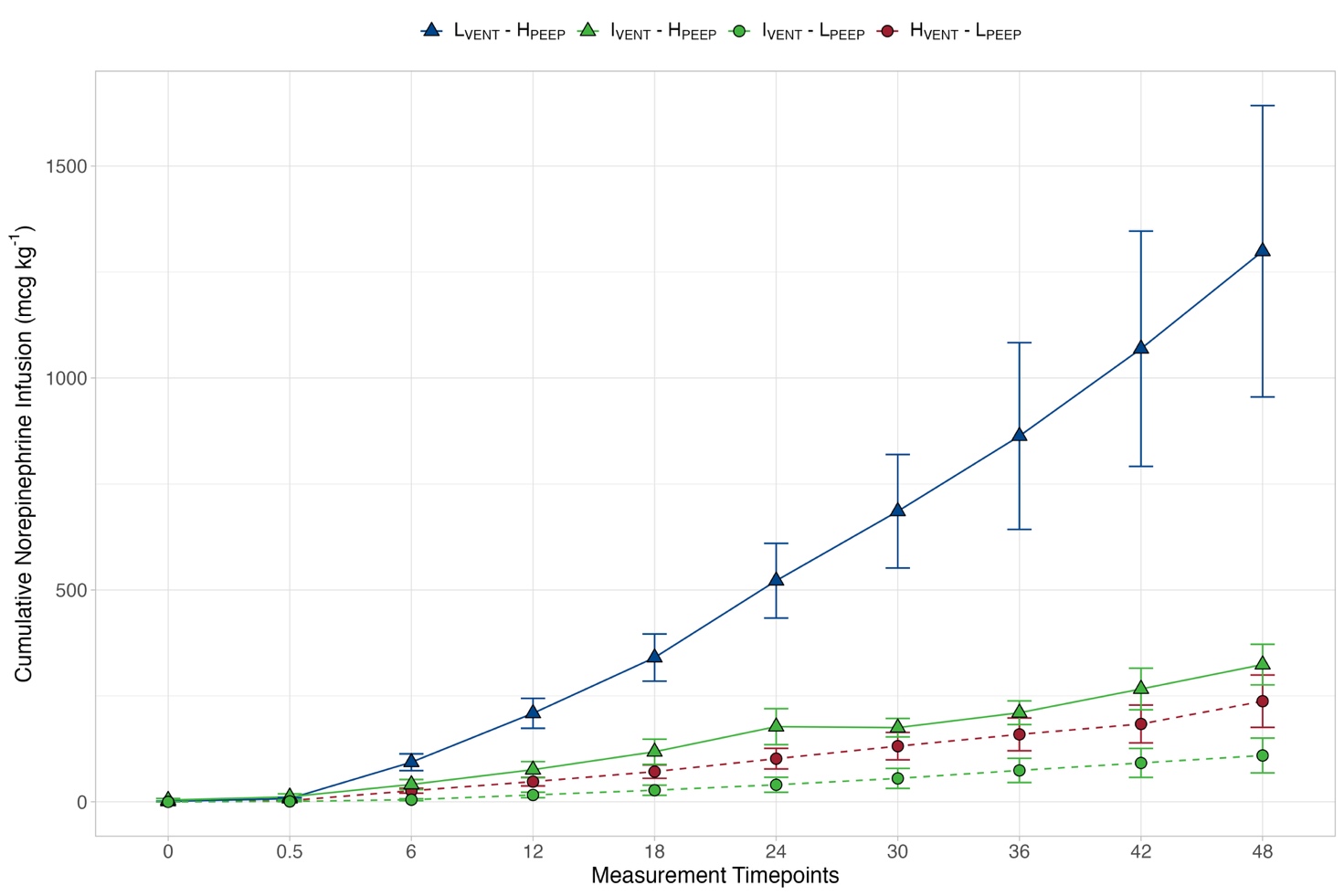

Supplement: Supplementary file 1 — Data S1: [file PHY2-12-e70042-s001.docx]
